# 5-Aminolevulinic acid promotes cell division and young fruit development by suppressing the PpARF9–PpGH3.1 auxin-conjugation module in peach

**DOI:** 10.1186/s43897-026-00262-7

**Published:** 2026-09-07

**Authors:** Xing Gan, Jianting Zhang, Longbo Liu, Liuzi Zhang, Mohsin Iqbal, Qingze Zhao, Liangju Wang

**Affiliations:** 1 College of Horticulture, Nanjing Agricultural University, Nanjing, 210095 China; 2 School of Life Science, Huaibei Normal University, Huaibei, 235000 China

**Keywords:** 5-Aminolevulinic acid (ALA), Cell cycle, Peach fruits, Phytohormone, PpARF9, PpGH3.1, RNA-seq

## Abstract

5-Aminolevulinic acid (ALA) is an emerging plant growth regulator, yet its mechanisms in fruit development remain unclear. Here, we show that ALA promotes early growth and ultimate size in peach (*Prunus persica*) fruits. ALA treatment significantly enhanced the mesocarp area and the cell number, accompanied by elevated levels of indole-3-acetic acid (IAA) and trans-zeatin riboside. Transcriptomic analysis identified PpARF9 as a key transcriptional factor, regulating auxin homeostasis and responsive to ALA treatment. Functional studies with yeast-one-hybrid (Y1H), dual-luciferase (DLR), electrophoretic mobility shift assay (EMSA) and homogenous genetic transformation revealed that PpARF9 directly transcriptionally activates *PpGH3.1*, a crucial gene which encodes chloroplastic auxin amide synthetase to conjugate free IAA and repress cell division. Overexpression of *PpGH3.1* reduced free IAA with more IAA conjugate accumulation, thereby suppressing cell cycle. Conversely, silencing *PpGH3.1* with RNA interference had opposite effects. Notably, exogenous ALA alleviated the inhibitory effects of *PpARF9* and *PpGH3.1* on cell proliferation. These findings indicate that ALA promotes cell division by downregulating the PpARF9–PpGH3.1 module to maintain higher levels of active IAA, facilitating cell division, increasing cell number and fruit size. These provide a mechanistic insight into ALA-regulated fruit development and support its application in improving fruit production.

**Supplementary Information:**

The online version contains supplementary material available at https://doi.org/10.1186/s43897-026-00262-7.

## Core

This study implemented an integrated approach that combined histo-anatomical phenotype, endogenous plant hormone quantification, transcriptome sequencing, and gene functional validation. We demonstrated that ALA downregulated the PpARF9-PpGH3.1 auxin-conjugation module, thereby sustaining elevated free IAA levels. This hormonal status enhanced mesocarp cell division and increased final cell number, leading to larger fruit with improved quality at harvest. Our findings establish the first molecular framework for ALA-mediated regulation of early fruit development, facilitating ALA application in boosting fruit market competitiveness.

## Gene & accession numbers

The reference genome used for RNA-seq alignment in this study was *P. persica* NCBIv2 (GCF_000346465.2), accessible at the NCBI repository (https://www.ncbi.nlm.nih.gov/datasets/genome/GCF_000346465.2/). After read mapping, we generated a complete annotation file containing gene-expression values. The accession numbers for the key genes analyzed are as follows: *PpARF9* (Gene ID: ncbi_18776765) and *PpGH3.1* (Gene ID: ncbi_18768891).

## Introduction

Peach (*Prunus persica* L.) is one of the most significant temperate deciduous fruit trees globally (Yu et al. 2019). According to the data of the Food and Agriculture Organization of the United Nations (FAO) in 2021, China accounted for 54.83% of the global peach planting area and 64.08% of the global peach production, ranking first in the world in both categories. Therefore, China is the main contributor to the global peach industry. However, fruit market competition is increasingly intensifying, which forces the growers to employ distinct cultivation techniques to increase fruit yield and improve fruit quality. The primary techniques to enhance fruit yield and quality mainly include germplasm improvement, new cultivation patterns to modify environments, water supply and fertilizer management, and application of growth regulators (Jiao 2009; Sourav And Sadawarti 2020). Among them, plant growth regulator application is comparatively swift, cost-effective, and efficient. Yet, the physiological and molecular mechanisms underlying are not fully understood, which restricts their application in practice.

5-Aminolevulinic acid (ALA) is an essential biosynthetic precursor of tetrapyrrole compounds (Wu et al. 2019). It is also recognized as a novel plant growth substance, involved in regulating plant growth and development (Wang et al. 2023a, 2025), such as root elongation growth (An et al. 2019), stomatal opening (Chen et al., 2023b, c; Liu et al. 2025a), leaf photosynthesis (Liang et al. 2023; Wang et al. 2024b), fruit coloration (Zheng et al. 2017), and biotic (Yang et al. 2024) as well as abiotic stress tolerance (Li et al. 2025b; Yang et al. 2025; Yuan et al. 2025; Zhong et al. 2025). In apple, ALA promotes apple anthocyanin accumulation (Wang et al. 2004), and the underlying mechanisms have been extensively studied in the past decade (Xie et al. 2013b; Fang et al. 2022; Zhang et al. 2024; Yin et al. 2025). Comparatively, the effect of ALA on fruit growth is seldom studied. Since the size is an important quality trait associated with both yield and market competitiveness, studies in this area are of considerable significance. In date palm, ALA promotes the fruit size (Al-Khateeb et al. 2006; Al-Qurashi and Awad 2011). In kiwifruit, ALA application before blossom can increase fruit sizes as well as flavor quality (Huang et al. 2021). However, in peach, ALA spraying five weeks after flower falling increased the content of the soluble sugars, vitamin C, with lower titratable acids of mature fruits, but not fruit mass (Ye et al. 2017). It seems that whether ALA can promote fruit sizes is elusive. And the underlying mechanisms that ALA regulates fruit development are unbeknown.

Phytohormones play a critical role in regulating young fruit development (Baral et al. 2025). Subsequent to pollination and fertilization, the set fruits begin to grow. A surge of auxin (indole-3-acetic acid, IAA) in the ovules stimulates early embryo development, which is the crucial prerequisite for young fruit growth (Robert et al. 2018). Cell division and volume expansion are the two distinct but intimately related aspects, where the former is primarily controlled by IAA (Fenn and Giovannoni 2021). In the proteasome-dependent IAA-signaling route, TRANSPORT INHIBITOR RESPONSE1/AUXIN-SIGNALING F-BOX (TIR1/AFB) is the co-receptor of IAA, while the auxin/indole-3-acetic acid (Aux/IAA) as the repressor and the auxin response factor (ARF) as the transcription factors (TFs) are involved (Martin-Arevalillo et al. 2025). When IAA is absent or at low levels, ARFs cannot bind to auxin response elements (AuxREs) of the auxin response gene promoters. Instead, ARFs form heterodimers with Aux/IAA, blocking transcription of auxin response genes. When IAA is increased, it can bind to TIR1/AFB, forming the auxin-TIR/AFB-Aux/IAA complex to trigger Aux/IAA ubiquitin-mediated degradation, releasing ARF repression and activating the downstream gene transcription (Gray et al. 2001; Karim et al. 2024). The working model suggests that the IAA level is crucial for auxin-mediated growth and development. In fact, the *Aux*/*IAA* family, the *ARF* family, the *small auxin upregulated RNA* (*SAUR*) family, and the auxin-responsive *Gretchen Hagen3* (*GH3*) family are all early auxin-response genes involved in the IAA regulating plant growth and development (Luo et al. 2018; Bao et al. 2024). Conversely, IAA can interplay with gibberellic acids (GAs) or other phytohormones, synergistically regulating fruit growth (Kumar et al. 2014; He and Yamamuro 2022). Cytokinins (CTKs) also foster cell division in the early fruit development, as reported in tomato (Gan et al. 2022) and woodland strawberry (Tian et al. 2022). In addition, abscisic acid (ABA) facilitates the unloading of assimilates through the phloem into young fruits, thereby supporting the early growth (Gupta et al. 2022). These indicate that numerous plant hormones are essential for the regulation of fruit growth.

The relationship between ALA and phytohormones in regulating plant growth and development has been proposed (Wang et al. 2025). In 1998, ALA was initially documented to have the dual hormonal function of IAA and cytokinin, because it can induce regeneration of adventitious roots as well as adventitious buds (Bindu and Vivekanandan 1998). In the roots of *Arabidopsis*, ALA upregulates *AtPIN1* expression, which is responsible for IAA polar transport and primary root elongation growth (An et al. 2019). In the guard cells of apple leaves, ALA antagonizes ABA-induced stomatal closure (Chen et al. 2023b; c; d). In the regulatory process, ALA induces an increase of protein phosphatase 2 A (PP2A) activity to dephosphorylate the sucrose non-fermenting-1-related protein kinase 2.6 (SnRK2.6, a component in ABA signal route), and block ABA-induced stomatal closure. Under polyethylene glycol (PEG)-mimicked drought stress, ALA-induced drought tolerance of strawberry may be mediated by jasmonic acid (JA), where ALA increases the (+)−7-iso-jasmonoyl-L-isoleucine content in leaves and roots. By contrast, diethyldithiocarbamate (DIECA), an inhibitor of JA biosynthesis, aggravates the adverse effects of PEG, while exogenous ALA relieves the adverse impact (Zhong et al. 2025). In red pear (*Pyrus ussuriensis* cv. ‘Nanhong’), exogenous ALA upregulates the expression of *PuNCED* (encoding 9-*cis*-epoxycarotenoid dioxygenase) to increase the endogenous ABA content and stimulate anthocyanin accumulation, while fluridone, an inhibitor of ABA biosynthesis can block ALA-induced anthocyanin accumulation (Cao et al. 2022). These findings suggest that ABA is essential for ALA to induce anthocyanin accumulation. However, the effect needs further verification. Overall, compelling evidence linking specific phytohormones to ALA-promoted fruit growth remains lacking.

Fruit growth is primarily dependent on cell division, called as the cell cycle, which consists of sequential G1 (Gap one, synthesis for RNA and ribosomes), S (synthesis for DNA as well as histone), G2 (Gap two, preparatory for mitosis), and M phase (mitosis). It is the core determinant of cell proliferation during fleshy fruit development. Its progression is tightly regulated by multiple conserved modules, with the core axis formed by cyclins (CYCs) and cyclin-dependent kinases (CDKs), along with key regulators including dimerization partner (DP), RETINOBLASTOMA-RELATED (RBR), E2F (a transcription factor), anaphase promoting complex/cyclosome (APC/C) and R1R2R3-type MYB (MYB3R) transcription factors (Shimotohno et al. 2021). Auxin precisely modulates this process: it promotes G1/S transition by enhancing CYCD-CDKA-mediated phosphorylation and ubiquitination of the repressive DPb-E2Fc heterodimer, while stabilizing the activating DPa-E2Fb heterodimer to drive cell cycle progression (Gombos et al. 2023). As a key ubiquitin ligase, APC/C regulates cell cycle phase transition via degrading CYCs and CDKs; its specific activator CCS52A controls mitotic arrest and the transition to endoreduplication cycles (Vanstraelen et al. 2009). Functional studies in horticultural crops further confirm the critical roles. For example, in tomato, *SlCDKA1* and *SlCDKB* genes play opposite roles in pericarp cell division, with their misexpression reducing pericarp cell layers (Czerednik et al. 2012). In apple, B-type CDKs and A/B-type cyclins positively regulate fruit cell division, while CDK inhibitors MdKRP4/5 exert inhibitory effects (Malladi et al. 2011). Up to now, studies on the cell cycle of peach fruit cells, especially promoted by ALA are rarely reported.

In order to explore the effects of ALA on young fruit development and the underlying mechanisms, we first examined the effects of 10 mg L^−1^ ALA treatments (including a rhizosphere application one week before blossom and a foliar spraying when more than 50% flowers opened) on 'Zhongyou 4' nectarine. We found that ALA markedly promoted the fruit size, especially at the 3rd to 5th week after ALA spraying, as well as flavor quality at maturation. Then, we compared the anatomic differences of the young fruits between the ALA treatment and the control collected from the 3rd to 5th week after ALA spraying, and found that the main differences were present in the cell number of the mesocarps, possibly related to cell division. We next quantified endogenous hormones in seeds and mesocarps from the 3rd to 5th week after ALA spraying, and found that auxin and cytokinin constituted the main differentially accumulated hormones between the ALA-treated and the control samples. Subsequently, we performed transcriptome analysis with the seeds and mesocarps, and paid attention to the differentially expressed genes (DEGs) in the IAA and CTK signal routes. Ultimately, we selected *PpARF9* and *PpGH3.1* as the key target genes, identified that PpARF9 is a negative transcription factor involved in ALA-promoted cell division in the mesocarps of peach fruits, whereas *PpGH3.1* is a downstream target gene of PpARF9, responsible for IAA conjugation. Both negatively regulated cell division as well as cell cycle-related gene expression, while exogenous ALA can suppress their effects. It is the first report showing that ALA promotes fruit cell division through the IAA signaling, offering novel functional genes for peach genetic modification as well as facilitating the better usage of ALA in high quality fruit production.

## Results

### ALA increased the cell number of mesocarps to promote the fruit size of peaches

**Fig. 1:**
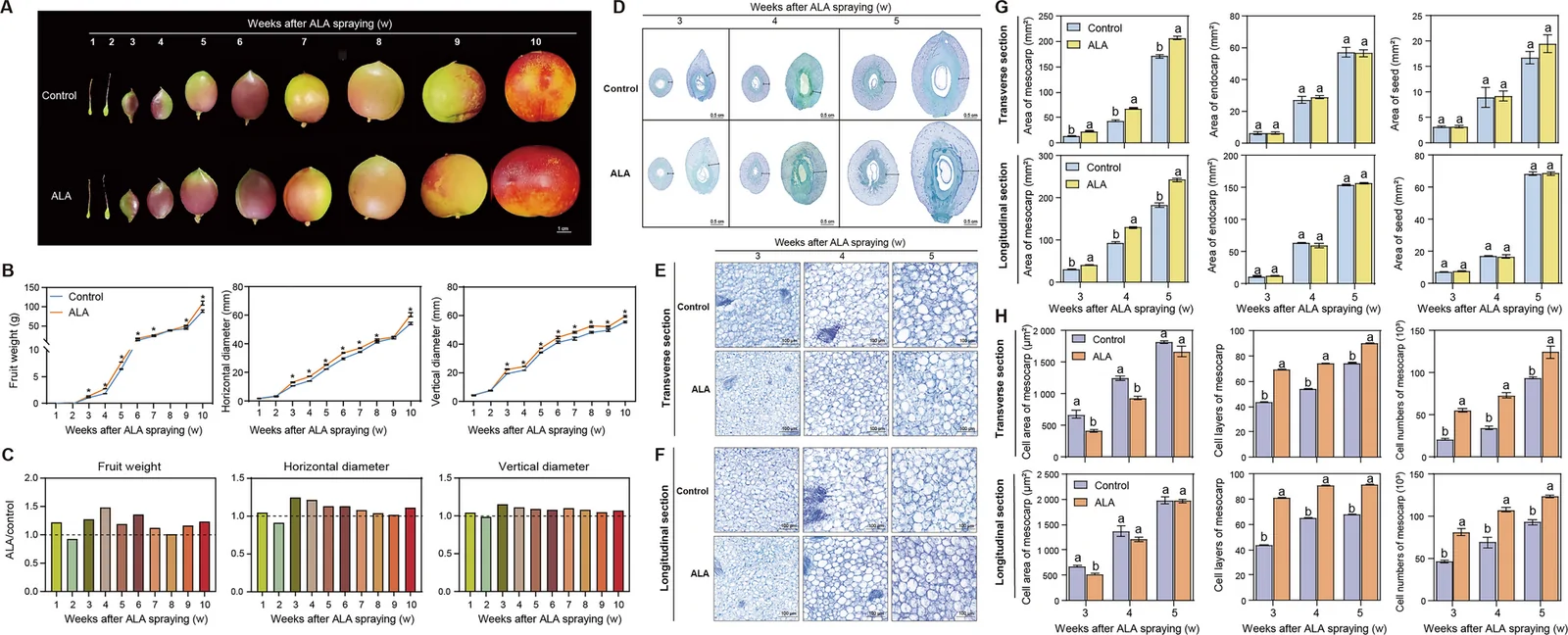
ALA enhances the size of fruits by increasing the cell number of the mesocarp. **A** Comparison of fruits between the control and ALA treatment after ALA spraying, the scale = 1 cm. **B** Comparison of single fruit weight, horizontal and vertical diameters after ALA spraying, respectively, where * indicates significant difference between the ALA treatment and the control at *P* = 0.05 (Student's *t*-test). **C** The relative values of single fruit weight, horizontal and vertical diameter between the ALA treatment and the control, respectively. The dash lines in the panels indicate 1.0. **D** The largest transverse (left) and longitudinal (right) sections of single fruit from the 3rd to 5th week after ALA spraying. The scale = 0.5 cm. **E–F** The local cells of the mesocarp of the peach fruit in the transverse and longitudinal sections from the 3rd to 5th week after ALA spraying. The scale = 100 μm. **G** The areas of the mesocarp, endocarp and seeds in the transverse and longitudinal sections of the peach fruit from the 3rd to 5th week after ALA spraying, respectively. The different letters at each time indicate significant difference between the ALA treatment and the control (Student's *t*-test *P* < 0.05). **H** The cell areas, layers, and numbers of the mesocarps in the transverse and longitudinal sections of peach fruits from the 3rd to 5th week after ALA spraying, respectively. The data are means ± standard error (SE) of more than three biological replicates, and the different letter indicates the significant difference at each time (Student's *t*-test *P* < 0.05)

To investigate the effect of ALA on the growth of peach fruits, we conducted weekly measurements of the young fruit sizes after ALA spraying (Fig. 1A). In the first two weeks, there were no significant differences observed between the ALA treatment and the control. After then, the fresh weight, the horizontal and vertical diameters of the fruits in the ALA treatment were significantly higher than that of the control (Fig. 1B). The relative values of the single fruit weight, especially the horizontal and vertical diameters from the 3rd to 5th week after ALA spraying were consistently higher than the control (Fig. 1C). These imply that ALA promoting fruit size mainly exhibited in this phase. When fruits were mature (ten weeks after ALA spraying), the single fruit weight of the ALA-treated was 22.92% higher than that of the control (*P* < 0.05, Fig. S1A). Meanwhile, the content of soluble sugars, soluble proteins, and vitamin C of the ALA-treated was significantly higher than that of the control (Fig. S1B). In addition, ALA had tendency to depress the titratable acid content of peach fruits, although not significant at *P* = 0.05, the ratio of sugar/acid was significantly increased after ALA treatment (*P* < 0.05). Collectively, ALA improved peach quality not only in the external appearance but also in the internal flavor.

Considering the enhancement of ALA on fruit size predominantly appeared in the 3rd to 5th week after ALA spraying, we analyzed the anatomical differences of the young fruits treated with ALA and the control. The results showed that the area of neither the endocarp nor the seed-including transverse and longitudinal sections was affected by ALA treatment; however, ALA significantly enlarged the mesocarp area of young fruits from the 3rd to 5th week after spraying (Fig. 1D and G). When the mesocarp cells were compared under a higher magnification microscope (Fig. 1E-H), we found that the single cell areas of the mesocarp in the 3rd week after ALA spraying were only 42.96% and 59.70% of the control in the transverse and longitudinal sections, respectively (*P* < 0.05), and the difference was still obvious in the 4th week in the transverse section but not in the longitudinal section. However, the cell layers and cell numbers in the mesocarp of the ALA-treated fruits were both significantly more than that of the control. In the 5th week, the cell layer and cell number in the transverse section of ALA-treated fruit mesocarps were 126.36% and 150.21% as high as that of the control, respectively, while that in the longitudinal section were 134.47% and 120.78% as high as the latter (*P* < 0.05). These data suggest that ALA treatment significantly promoted cell division of mesocarp with increased cell layer and cell number of peach fruits, although the single cell area was much smaller than that of the control at the early stage, especially in the transverse section. When the cells went through the expansion stage, the single cell areas of mesocarps of the ALA-treated fruits became as large as those of the control. Therefore, ALA-promoting cell division to increase the cell numbers in the mesocarps of peach fruits was responsible for the larger size when they were mature (Fig. S1A).

### ALA affected the phytohormone levels in the seeds and mesocarps of young peach fruits

**Fig. 2:**
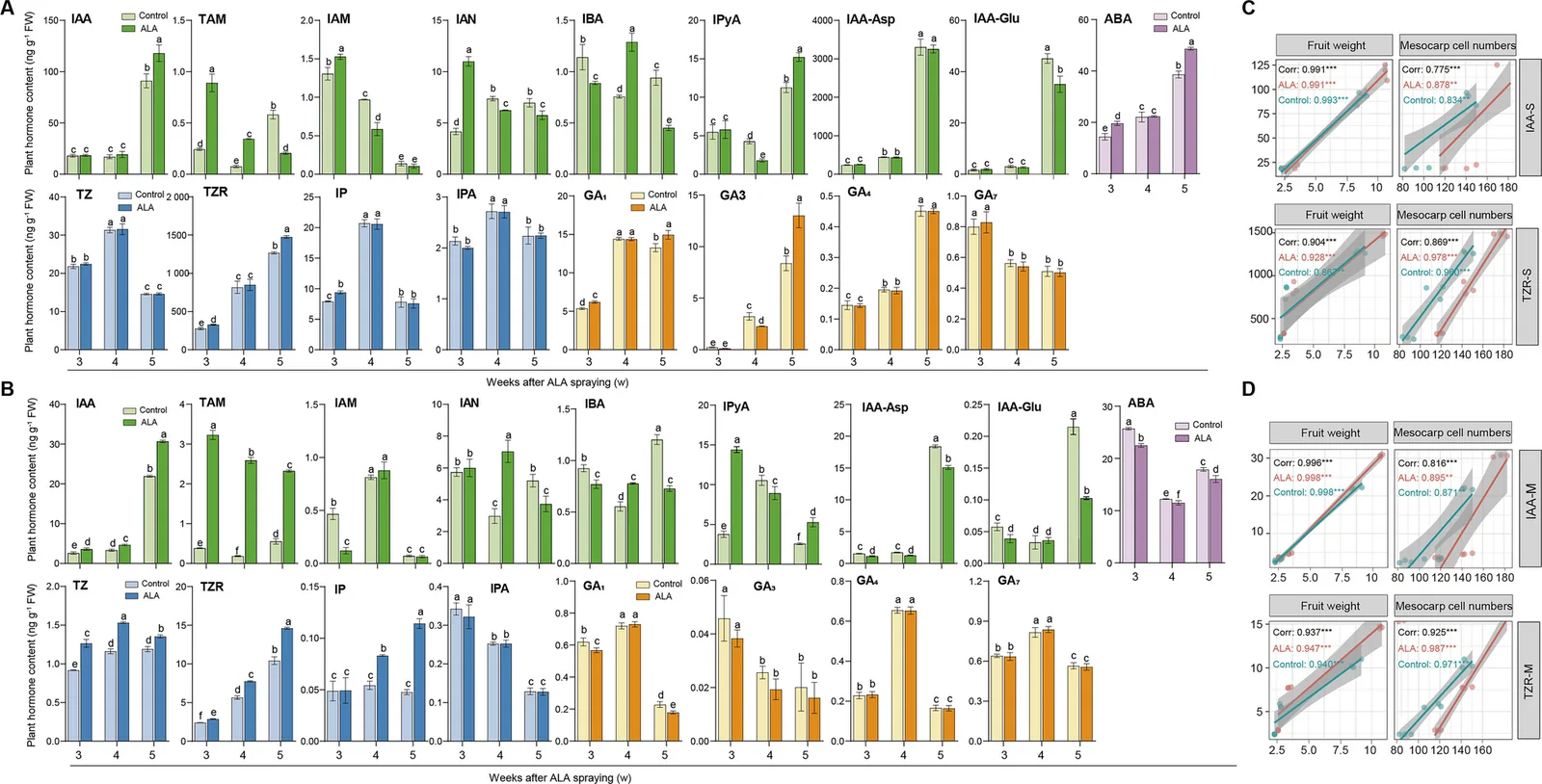
The plant hormone content from the 3rd to 5th week after ALA spraying. **A-B** Detection of hormone content in the seed (**A**) and mesocarp (**B**). The content of eight compounds related with auxins (indole-3-acetic acid, IAA; tryptamine, TAM; indole-3-acetonitrile, IAN; indole-3-acetamide, IAM; indole-3-butyric acid, IBA; indole-3-pyruvic acid, IPyA; aspartate conjugate of indole-3-acetic acid, IAA-Asp; and glutamine conjugate of indole-3-acetic acid, IAA-Glu), four compounds related with cytokinin (trans-zeatin, TZ; trans-zeatin riboside, TZR; isopentenyladenine, IP; and isopentenyladenosine, IPA), four active gibberellins (GA_1_, GA_3_, GA_4_, GA_7_), and abscisic acid (ABA). The columns filled with the similar color represent the same kind of phytohormone. **C-D** Correlation of fruit weight and mesocarp cell numbers with the content of IAA and TZR in the seeds (**C**) and mesocarps (**D**). The data are from three independent biological replicates, and the values are means ± SE (n = 3). The same letter in each panel indicates no significant difference at *P* = 0.05. Asterisks '**' and '***' indicate significance at *P* = 0.01 and 0.001, respectively. The abbreviations IAA-S and IAA-M indicate IAA levels in seed and mesocarp, respectively; similarly, TZR-S and TZR-M denote TZR concentrations in these tissues

Since young seeds are considered as the main sites for phytohormone biosynthesis, we quantified the content of the endogenous indole-3-acetic acid (IAA), as well as its biosynthetic precursors (including tryptamine, TAM; indole-3-acetonitrile, IAN; indole-3-acetamide, IAM; indole-3-butyric acid, IBA; and indole-3-pyruvic acid, IPyA) and the metabolites (including aspartate conjugate of indole-3-acetic acid, IAA-Asp; and glutamine conjugate of indole-3-acetic acid, IAA-Glu) in the seeds of peach fruits from the 3rd to 5th weeks after ALA spraying. The results showed that the IAA content was much higher than its precursors, suggesting it is the main product in the seeds of peach. However, it kept at relative low levels in the 3rd to 4th week after ALA spraying, but increased more than 5 times in the 5th week. Furthermore, ALA significantly increased the IAA content at this time, reaching 129.58% as high as that of the control (*P* < 0.05). As for the biosynthetic precursors of IAA, their content in the 3rd to 5th week, at least one time in the ALA treatment was significantly higher than that of the control. These suggest that ALA promoted IAA biosynthesis, although the data was unstable during the seed development (Fig. 2A). On the other hand, the content of IAA-Asp and IAA-Glu, especially the former was much higher than that of the precursors. And, the content of IAA-conjugates was highly correlated with that of endogenous IAA. In the 3rd and 4th week, they were relatively low but dramatically increased in the 5th week after ALA spraying. ALA treatment did not affect the content in the first two weeks, but significantly depressed the increases in the 5th week, where the difference in IAA-Glu was significant (*P* < 0.05). These data imply that the biosynthesized IAA is rapidly conjugated into the conjugate compounds, while ALA inhibits IAA conjugation especially with glutamic acid. Consequently, ALA promotes IAA biosynthesis and depresses IAA catabolism in the seeds of peaches. The bidirectional effect facilitates increased active auxin levels.

Furthermore, ALA significantly increased the content of trans-zeatin riboside (TZR) and isopentenyladenine (IP) in the seeds of fruits after ALA spraying for 3 to 5 weeks (Fig. 2A). However, no significant differences in the content of trans-zeatin (TZ) and isopentenyladenosine (IPA) were observed between the ALA treatment and the control. Likewise, ALA significantly increased the content of gibberellic acid (GA_1_) and GA_3_, but had no significant effect on GA_4_ and GA_7_ in the peach seeds. In addition, ALA increased the ABA content in the seeds of peach fruits after spraying 3 to 5 weeks. Correlation analysis (Fig. 2C) showed that only the content of IAA and TZR exhibited strong positive correlations with fruit weight as well as cell number of mesocarps (*P* < 0.01 or 0.001), while no significant correlations were observed for the other hormones. These imply that ALA-promoted fruit size as well as cell number of peach may be mediated by enhanced auxin and cytokinin levels in the peach seeds.

In the mesocarps, most phytohormone levels were significantly lower than those in the seeds. Among them, the levels of auxins (such as IAA, as well as its metabolites IAA-Asp and IAA-Glu) and cytokinins (including TZ and TZR) in the mesocarp steadily increased from 3 to 5 weeks after ALA spraying, while the other hormone content varied without discernible trends (Fig. 2B). Nonetheless, ALA treatment remarkably increased the auxin and cytokinin content, but tended to decrease the GA and ABA content. Especially in the ABA, the content within the ALA-treated mesocarp was significantly lower than that of the control. Conversely, ALA increased the auxin content, among which, ALA-induced TAMs showed the greatest increase. The overall mean in the mesocarps of ALA-treated fruits was 7.28 times as high as that of the control. Other species of auxins including IAA, IAN, and IPyA were also significantly higher than that of the control at different time points. Conversely, ALA obviously decreased the IAA-Asp and IAA-Glu content in the mesocarps. It once again suggests that ALA enhances IAA production meanwhile inhibits the degradation. In cytokinins, ALA increased the content of TZ, TZR and IP (*P* < 0.05), except IPA. Therefore, ALA has a positive effect on the auxin and cytokinin content but negative on ABA in the mesocarp during young fruit development. Correlation analysis showed that the IAA and TZR content in the mesocarp were significantly related to fruit weight and mesocarp cell number (*P* < 0.01 or 0.001) (Fig. 2D), but no close correlation was found in the other hormone compounds.

### Transcriptomic analysis of peach seeds and mesocarps after ALA spraying

We conducted transcriptome analysis on seeds and mesocarps collected from the 3rd to 5th week post-ALA spraying to investigate differentially expressed genes (DEGs) during ALA-promoted peach fruit development. More than 37 million paired-end raw reads were obtained among all the 36 samples, in which approximately 99.5% were kept as clean reads after filtering out the adapter and low-quality reads. The clean reads were uniquely mapped to the reference genome (*P. persica* NCBIv2) for read counts and FPKM calculation. A total of 24,236 genes were identified in all sequencing samples, including 1101 novel genes (Table S1). Eight DEGs were randomly selected for RT-qPCR validation. Pearson's correlation coefficients of RT-qPCR and RNA-Seq results were all higher than 0.991 (*P* < 0.001, Fig. S2), affirming the reliability of the RNA-Seq data. Among the gene expression profiles, we identified *PpARF9* (ncbi_18776765), whose expression was significantly downregulated by ALA in both seeds and mesocarps.

**Fig. 3:**
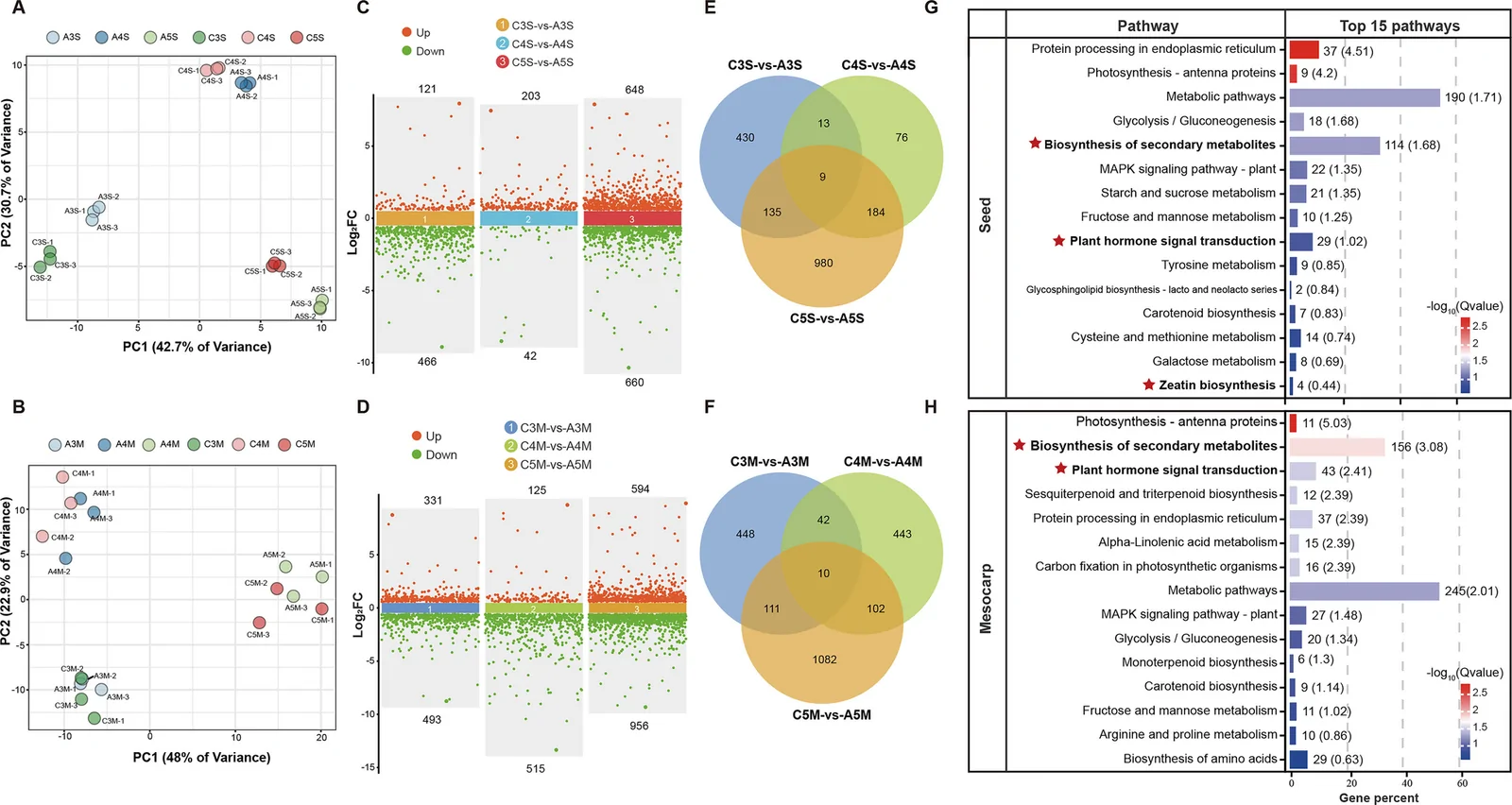
Transcriptomic analysis of the seeds and mesocarps of peach fruits after ALA spraying for 3rd to 5th week. **A-B** Principal component analysis of the three samples in the seeds and mesocarps, respectively. Every dot is one sample, and the closer the distribution of samples with same color, the better the biological reproducibility. S represents seeds; M represents mesocarps. 3, 4, 5 represent the 3rd, 4th and 5.^th^ week after ALA spraying. **C-D** The multi-group differential scatter plot of differentially expressed genes (DEGs) selected according to the criteria of |FC|> 1.5 and *P*-value < 0.05. FC (fold change) represents FPKM-ALA/FPKM-Control. **E–F** Vann diagrams of DEGs between control and ALA in the seeds and mesocarps, respectively; **G-H** KEGG enrichment of all DEGs between control and ALA in the seeds and mesocarps, respectively. FDR is the abbreviation of False Discovery Rate. The color bar represents enrichment significance, and the redder the color in the legend, the more significant the pathway enrichment. The pathways related with phytohormones are marked with red five stars. The description of KEGG pathways is listed in Table S3

Principal component analysis indicates that three biological replicates exhibited considerable repeatability (Fig. 3A and B), confirming the suitability for subsequent analysis. It can be seen that the numbers of DEGs in the mesocarp were higher than those in the seed, and the downregulated DEGs were higher than the upregulated genes (Fig. 3C and D). The total DEG numbers in the mesocarp and seed were 2238 and 1827, respectively, and the common DEGs of three time points were 10 and 9, respectively (Fig. 3E and F, Table S2).

To further understand the impact of ALA on the young fruit development, KEGG pathway enrichment analysis was conducted with all the DEGs identified in the mesocarps and seeds. The top fifteen pathways significantly enriched (FDR < 0.05) are listed in Fig. 3G and H. In the seed, the pathways related to phytohormones such as 'Plant hormone signal transduction' (ko04075) and 'Zeatin biosynthesis' (ko00908) were significantly enriched. In the mesocarp, 'Plant hormone signal transduction' was also significantly enriched. Additionally, 'Photosynthesis-antenna proteins' (ko00196), 'Protein processing in endoplasmic reticulum' (ko04141), 'Biosynthesis of secondary metabolites' (ko01110) were significantly enriched in seeds or mesocarps. These results confirm that ALA treatment considerably affected phytohormone signals in young peach fruits, possibly including hormone metabolism and signal transduction.

### Correlation analysis of the DEGs in the transcriptome with the IAA and TZR content

**Fig. 4:**
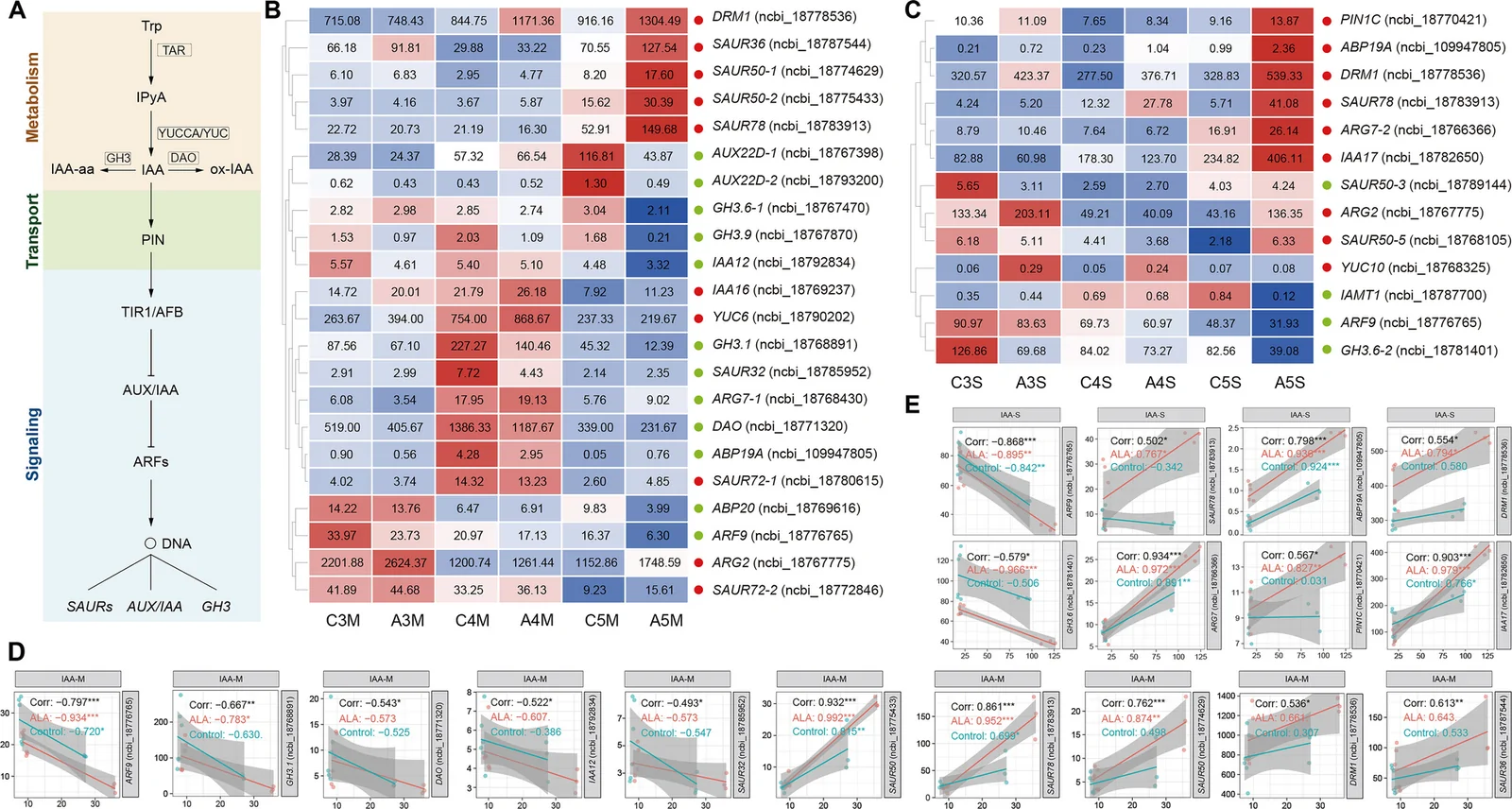
Analysis of the correlation between IAA and DEGs related to auxin. **A** The routes of auxin metabolism, transport, and signal transduction. Arrows indicate the subsequent metabolites at each pathway step; flat-headed arrows denote inhibition. Enzymes catalyzing rate-limiting reactions are boxed and comprise tryptophan aminotransferase-related proteins (TAR), the flavin-containing monooxygenase (YUCCA/YUC) family, IAA-amino synthetases (Gretchen Hagen 3, GH3), and DAO (dioxygenase for auxin oxidation). IAA-amino acid conjugates (IAA-aa) and oxidized IAA (oxindole-3-acetic acid, oxIAA) are the covalent adduct and oxidative catabolite of indole-3-acetic acid (IAA), respectively. PIN-FORMED (PIN) family polar auxin efflux carriers mediate directional auxin transport, whereas the TIR1/AFB F-box receptor complex perceives intracellular IAA levels. AUX/IAA (auxin-induced protein) and auxin response factors (ARFs) together constitute the core signaling module that orchestrates transcriptional activation or repression of downstream targets, including *SAUR* genes, additional *AUX*/*IAA*, and *GH3*. **B-C** Heatmaps of DEG expression (comparison between the ALA treatment and the control) in the mesocarps and the seeds, respectively. The right y-axis labels of the heatmap are presented in the format “gene symbol (gene ID),” both derived from the *Arabidopsis* reference-genome annotation. When multiple genes share the same symbol, they are distinguished in top-to-bottom order by appending an Arabic numeral immediately after the symbol with a non-breaking hyphen (e.g., *SAUR50-1*, *SAUR50-2*). The original gene IDs remain within the parentheses to ensure full traceability. The values are the means of FPKM of 3 biological replicates. The upregulated DEGs are marked with red circles, while the downregulated with green circles. **D-E** Correlation analysis between IAA content and the DEGs related to IAA signals in the mesocarps and the seeds, respectively. Asterisks '*', '**', and '***' indicate significance levels at *P* = 0.05, 0.01, and 0.001, respectively. The abbreviations IAA-S and IAA-M indicate IAA levels in seed and mesocarp, respectively

**Fig. 5:**
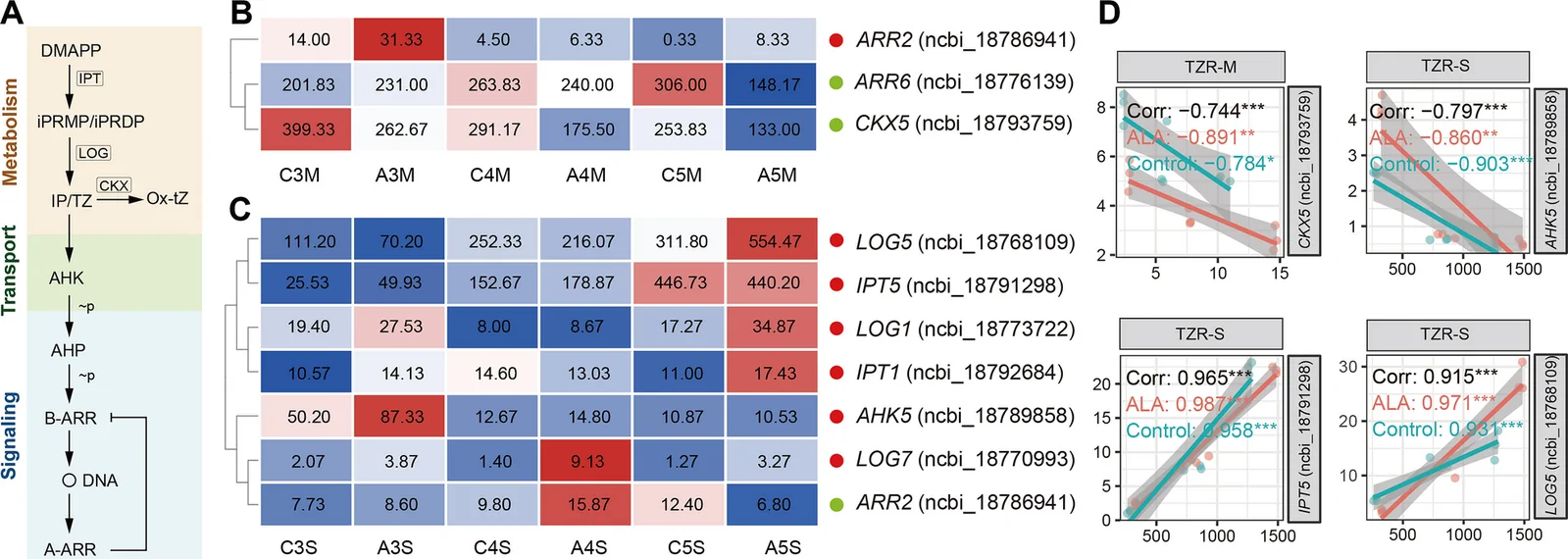
Analysis of the correlation between TZR and DEGs related to cytokinin. **A** Cytokinin metabolism, transport, and signal transduction pathway. The isoprenoid precursor dimethylallyl diphosphate (DMAPP) condenses with ATP/ADP under catalysis by isopentenyl transferase (IPT) to generate isopentenyladenosine-5'-mono- or diphosphate (iPRMP/iPRDP). Cytokinin riboside 5'-monophosphate phosphoribohydrolase (LONELY GUY, LOG) then hydrolyzes these nucleotides in a single step to release the bioactive free bases isopentenyladenine (IP) or trans-zeatin (TZ). Active cytokinins (CTKs) are irreversibly cleaved by cytokinin oxidase/dehydrogenase (CKX) to form inactive derivatives such as Ox-tZ, thereby maintaining intracellular CTK homeostasis. In the signaling branch, the membrane-anchored *ARABIDOPSIS HISTIDINE KINASE* receptor (AHK) perceives extracellular CTKs. Through a two-component phosphorelay cascade, the signal is transduced to cytoplasmic shuttling AHP proteins. Phosphorylated AHP (AHP ~ P) enters the nucleus and activates type-B ARR (B-ARR) transcription factors. Concurrently, type-A ARRs (A-ARRs) are rapidly induced as negative-feedback repressors to attenuate B-ARR activity, establishing a self-regulating loop. The symbol ~ P denotes phosphorylation. **B-C** Heatmaps of the DEG expression (comparison between the ALA treatment and the control) in the mesocarps and seeds, respectively. The upregulated DEGs were marked red circle side, while downregulated ones were marked green circle. **D** Correlation analysis between TZR and DEGs. The value represents FPKM (RNA-seq) means from 3 samples. Asterisks '*', '**', and '***' indicate significance levels at *P* = 0.05, *P* = 0.01, and *P* = 0.001, respectively. The abbreviations TZR-S and TZR-M denote TZR levels in seed and mesocarp, respectively

The findings in previous sections indicate the IAA and TZR content is highly correlated with fruit weight and mesocarp cell numbers, therefore they may be responsible for ALA-promoted fruit development. Utilizing the transcriptome data, we screened a batch of DEGs related to auxins (Fig. 4A) and cytokinins (Fig. 5A) including their metabolism, transport and signal transduction in the mesocarps and seeds. Figure 4B list the DEGs related with IAA in the mesocarp. From it, the dormancy-associated gene 1 *PpDRM1* was consistently upregulated during fruit development, which was further promoted by ALA treatment. Indole-3-pyruvate monooxygenase gene *PpYUC6*, involved in IAA biosynthesis, was upregulated, while 2-oxoglutarate-dependent dioxygenase gene *PpDAO*, responsible for auxin oxidation, was downregulated by ALA in the mesocarps. These indicate that ALA treatment promotes IAA synthesis and prevents oxidative inactivation. In addition, many DEGs in the mesocarps can be subordinated to early auxin response genes, including auxin-responsive protein *SAURs*, auxin response factor *ARFs*, indole-3-acetic acid-amido synthetase *GHs* and auxin-induced protein *IAA*/*AUX*. Several *SAURs* (such as 36/50/78) were upregulated by ALA, while *ARFs* (such as *ARF*9), *GHs* (such as 3.1/3.6/3.9) and *IAA*/*AUX* (such as 12/22D) were downregulated (Fig. 4B). Correlation analysis illustrates that IAA was significantly positively correlated with *PpSAUR50-1*/*50–2*/*78* but negatively with *PpARF9/PpGH3.1*, all correlation coefficients were higher in the ALA treatment than those in the control (Fig. 4D).

Similarly in the seeds, numerous DEGs associated with auxins were found, although the numbers were fewer than those observed in the mesocarp. Among the DEGs, auxin efflux carrier gene *PpPIN1C*, auxin-binding protein gene *PpABP19A*, and dormancy-associated gene 1 *PpDRM1* were upregulated by ALA. *PpYUC10* and *PIN1C* were upregulated by ALA. It is noteworthy that *PpARF9* was downregulated by ALA (Fig. 4C), which is consistent with that in the mesocarp. Analysis of correlation showed that IAA was significantly negatively correlated with *PpARF9/PpGH3.6* expression, whose correlation coefficients was higher in the ALA treatment than in the control (Fig. 4E). Therefore, the DEGs such as *PpARF9* may be important genes in ALA-regulated young peach fruit development.

The pathway of cytokinin synthesis, transport and signaling transduction is listed in Fig. 5A. From Fig. 5B and C, it is showed that the DEGs related to CTKs induced by ALA were much less than those of IAA, where only three DEGs found in the mesocarp, and seven in the seed. In the mesocarp, ALA depressed the cytokinin oxidase/dehydrogenase gene *PpCKX5*, whose expression was significantly negatively correlated with the TZR content (Fig. 5D). These suggest that ALA may suppress CTK metabolism to promote CTK accumulation. Two *ARABIDOPSIS RESPONSE REGULATORs* (*PpARR2*/6) were found regulated by ALA, where *PpARR2* expression was upregulated, while *PpARR6* was downregulated. However, both of them had no significant correlation with the TZR content, indicating lack of association with ALA-promoted mesocarp growth.

In the peach seeds, more DEGs related to cytokinin synthesis were found, including cytokinin riboside 5'-monophosphate phosphoribohydrolase genes (*LONELY GUY, LOGs*), isopentenyl transferase gene (*IPTs*), *ARABIDOPSIS HISTIDINE KINASE* gene (*AHK*), and *PpARR2* (Fig. 5C). *PpLOG1/5/7* were generally upregulated by ALA, so were *PpIPT1*/*5*. In addition, the expression of *PpAHK5* (encoding cytokinin receptor) was considerably upregulated in the 3rd week after ALA spraying, which was negatively correlated with TZR content changes (Fig. 5D). These findings indicate that ALA significantly influences cytokinin homeostasis by increased biosynthesis (through *PpIPT1*/*5* and *PpLOG1*/*5*/*7*), depressed metabolism (through *PpCKX5*) and increased signaling transduction (through *PpAHK5* and *PpARR2*).

### ALA eliminated the negative role of PpARF9 on cell division in peach callus

From the results above, we found that IAA signaling was closely related with fruit development, among which, the *PpARF9* expression was significantly downregulated by ALA (Fig. 4B and C), and negatively correlated with the IAA content in both the mesocarps and seeds (Fig. 4D and E). Therefore, PpARF9 may be an important negative factor responsive to ALA treatment, involved in ALA-promoted peach fruit growth, especially in mesocarp cell division (Fig. 1H). Then, we decided to identify functions of *PpARF9* in the subsequent study.

**Fig. 6:**
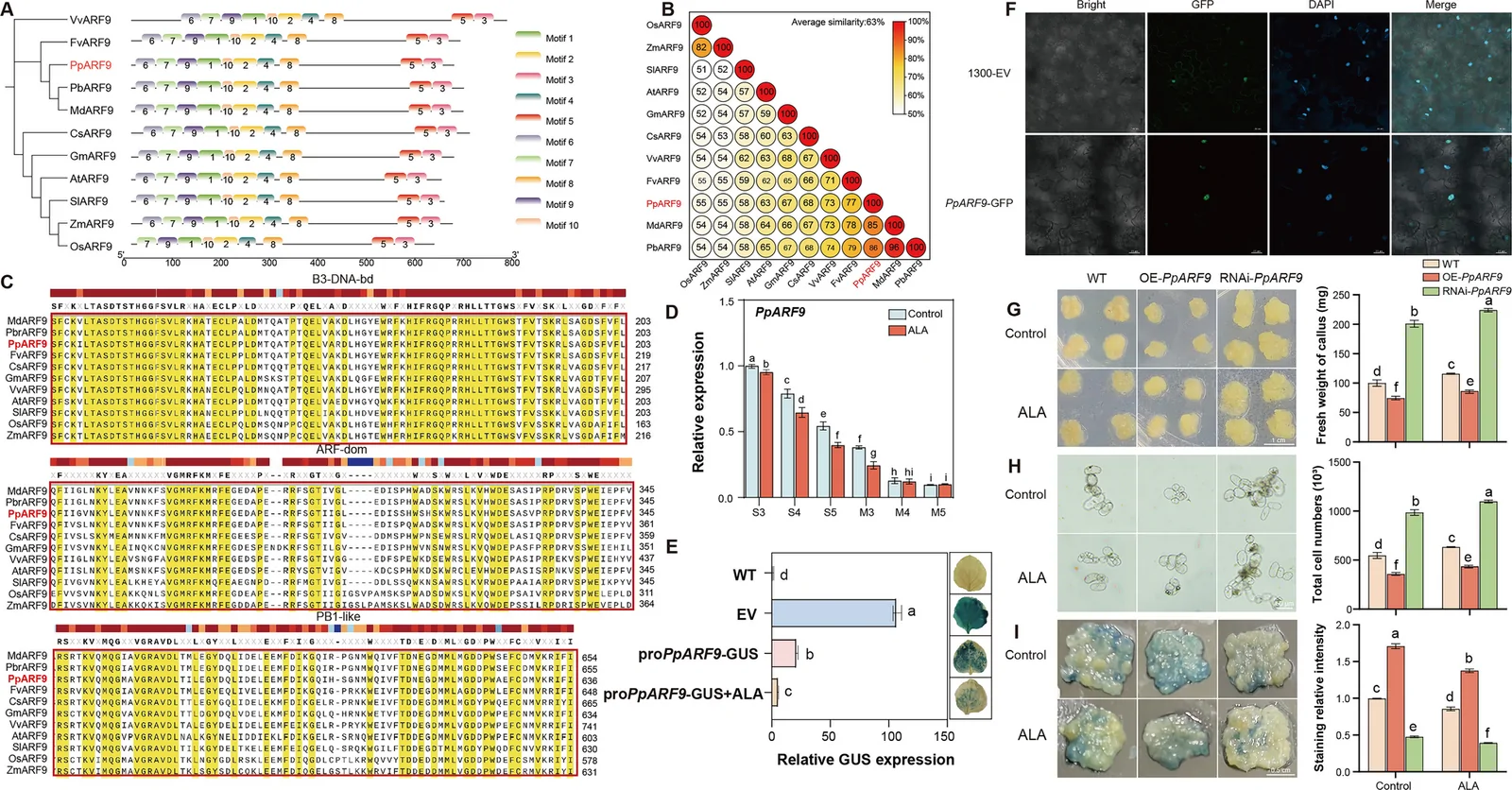
Identification of function of PpARF9 in ALA-regulated cell division in peach callus. **A** The phylogenic tree of the orthologous of PpARF9 and the conserved motif distribution. Here, eleven species are used, including peach (*P. persica*), apple (*Malus* × *domestica*), pear (*Pyrus bretschneideri*), woodland strawberry (*Fragaria vesca*), grape (*Vitis vinifera*), cucumber (*Cucumis sativus*), *Arabidopsis* (*Arabidopsis thaliana*), tomato (*Solanum lycopersicum*), soybean (*Glycine max*), rice (*Oryza sativa*), and maize (*Zea mays*). The ten conserved motifs are marked with different numbers and colors. **B** The sequence similarity analysis of PpARF9 orthologous. The numbers and colors within circles indicate the percentage of similarity. **C** Multiple sequence alignment among PpARF9 and its 10 orthologs in different species. The color legend above the alignments indicates the similarity of amino acid sequences at each position. The deeper the color, the higher the similarity. In the alignment arrays, the yellow shades indicate the consensus sequences. The red boxes indicate three conserved domains: B3-DNA binding domain (B3-DNA-bd), ARF dom (ARF-domain), and Phox-Bem1p (PB1-like) domain, respectively. **D** Relative expression of *PpARF9* in the seeds and mesocarps from the 3rd to 5th week after ALA spraying. The data are means ± SE of three biological replicates, and the same small letters indicate no significant difference at *P* = 0.05. **E** Effects of ALA on the transcriptional activity of *PpARF9* promoter by GUS staining method. The pro*PpARF9*-GUS vector (with the empty vector as the control) was transiently injected into tobacco leaves treated with 0.5 mg L^−1^ ALA or not. Then, the GUS staining was performed and the relative expression of *GUS* gene was measured. The measurement was conducted with three biological replicates, and the different small letters indicate significant difference at *P* = 0.05. **F** Subcellular localization of PpARF9. DAPI (4',6-diamidino-2-phenylindole) was used to mark nuclear. Scale bar = 20 μm and EV is the empty vector of pCambia-1300. **G** Callus phenotypes of 'ZJB' peach, which was stably transformed with overexpression (OE-*PpARF9*) and RNA interference (RNAi-*PpARF9*) vectors, cultured on the solid MS media for 15 d, containing 1 mg L^−1^ ALA or not. The scale = 1 cm. The positive identification of the transgenic cells by PCR and RT-qPCR was in Fig. S3. The fresh weights of callus are listed in the right histogram. **H** Observation of cell suspension under a microscope (the scale = 50 μm) and the cell numbers are listed in the right histogram. **I** Detection of cell viability by Evans blue staining, and relative intensity is listed in the right histogram. The data are means ± SE of three independent replicates. The different small letters in each panel represent significant differences at *P* = 0.05

Sequence analysis across various species indicates that ARF9 is highly evolutionarily conserved (Fig. 6A and B). In the species of *Rosaceae* (such as apple, pear, peach, and woodland strawberry), the average similarity is higher than 80.2%; even in the other species such as cucumber, tomato, *Arabidopsis*, soybean, rice and maize, the average similarity exceeds 62.7%. It seems that the closer the kinship, the higher the similarity of ARF9 protein sequences. Multiple sequence alignment reveals that ARF9 from these species harbor three canonical domains: B3-DNA binding (B3), ARF (ARF domain), and PB1-like functional domain (Fig. 6C). RT-qPCR results showed that the expression levels of *PpARF9* were decreased in the seeds and mesocarps of peach fruits from the 3rd week to 5th week after ALA spraying (Fig. 6D). Figure 6E showed that ALA treatment inhibited the transcriptional activity of *PpARF9* promoter, because the relative expression of pro*PpARF9*-GUS treated with ALA was significantly reduced compared to that of the control, and the intensity of GUS staining was also weakened after ALA treatment. Furthermore, the subcellular localization results (Fig. 6F) indicated that PpARF9 was located in the nucleus. These indicate that PpARF9 may act as a transcription factor, negatively responsive to ALA, functioning in the ALA-regulated fruit development.

We transformed the overexpressing (OE-) and RNA interfering (RNAi-) *PpARF9* vectors into peach callus. After verification by PCR and RT-qPCR (Fig. S3), the fresh cells were transferred to new MS solid media containing 1 mg L^−1^ ALA or not. Following a fortnight culture, the size and fresh weight of OE-*PpARF9* callus were significantly lower than that of the wild type (WT), whereas RNAi-*PpARF9* greatly promoted callus growth. ALA treatment significantly increased the callus size and fresh weight across all genotypic lines (Fig. 6G). These suggest that *PpARF9* inhibits cell proliferation, whereas ALA ameliorates the negative role of *PpARF9*. Furthermore, we transferred the callus clumps into MS liquid media and gently dispersed them into cell suspension. Under a microscope, it can be seen that the cell number in the OE-*PpARF9* was significantly lower than that of the WT, while the most was in the RNAi-*PpARF9* (Fig. 6H). In the ALA-treated callus, the cell number in all samples were increased. These suggest that PpARF9 depresses cell proliferation and decreases cell number of peach callus, while ALA treatment can ameliorate the inhibition of PpARF9. In addition, we employed the Evans blue method to detect the cell viability, as a deeper hue indicates less cell viability. As illustrated in Fig. 6I, compared with WT, OE-*PpARF9* decreased peach callus cell viability, while RNAi-*PpARF9* enhanced the cell viability, and ALA treatment improved the cell viability in all genotypic callus.

To verify the link between PpARF9 and cell division, RT-qPCR was conducted to detect the gene expression related to cell cycle (Fig. S4A). Compared with the WT, the majority of cell-cycle genes, such as *PpDPa*/*b*, *PpCCSA52A1*/*2*, *PpCYCA3-2*, *PpCYCD3-3*/*4–1*/*6–1*, and *PpCDKA*/*B2,* were significantly downregulated expression in OE-*PpARF9* callus, whereas *PpRBR* and *PpMYB3R3* were markedly upregulated. An opposite expression pattern for these genes was observed in the RNAi-*PpARF9* callus. These results indicate that *PpARF9* is intimately associated with cell cycle control and the related gene expression. ALA significantly elevated the expression of most cell cycle genes, such as *PpE2F*, *PpDPa*, *PpCCSA52A1*, *PpMYB3R1*, *PpCYCAs*, *PpCYCBs*, *PpCYCD3-3*/*6–1*, and *PpCDKA*/*B2,* and concomitantly repressed *PpRBR* and *PpMYB3R3* across all genotypes (*P* < 0.05). These indicate that ALA treatment improved cell cycle and cell proliferation by depressing the *PpARF9*-mediated gene expression. *PpDPa*, *PpCCSA52A1*, *PpRBR*, *PpMYB3R3*, *PpCYCD3-3*/*6–1* and *PpCDKB2* might be the key genes that respond to ALA as well as *PpARF9*. Collectively, *PpARF9* is a negative factor responsible for cell cycle and cell proliferation, which is negatively regulated by ALA.

### *PpGH3.1* as a target gene of PpARF9 negatively regulates ALA-promoted cell division

**Fig. 7:**
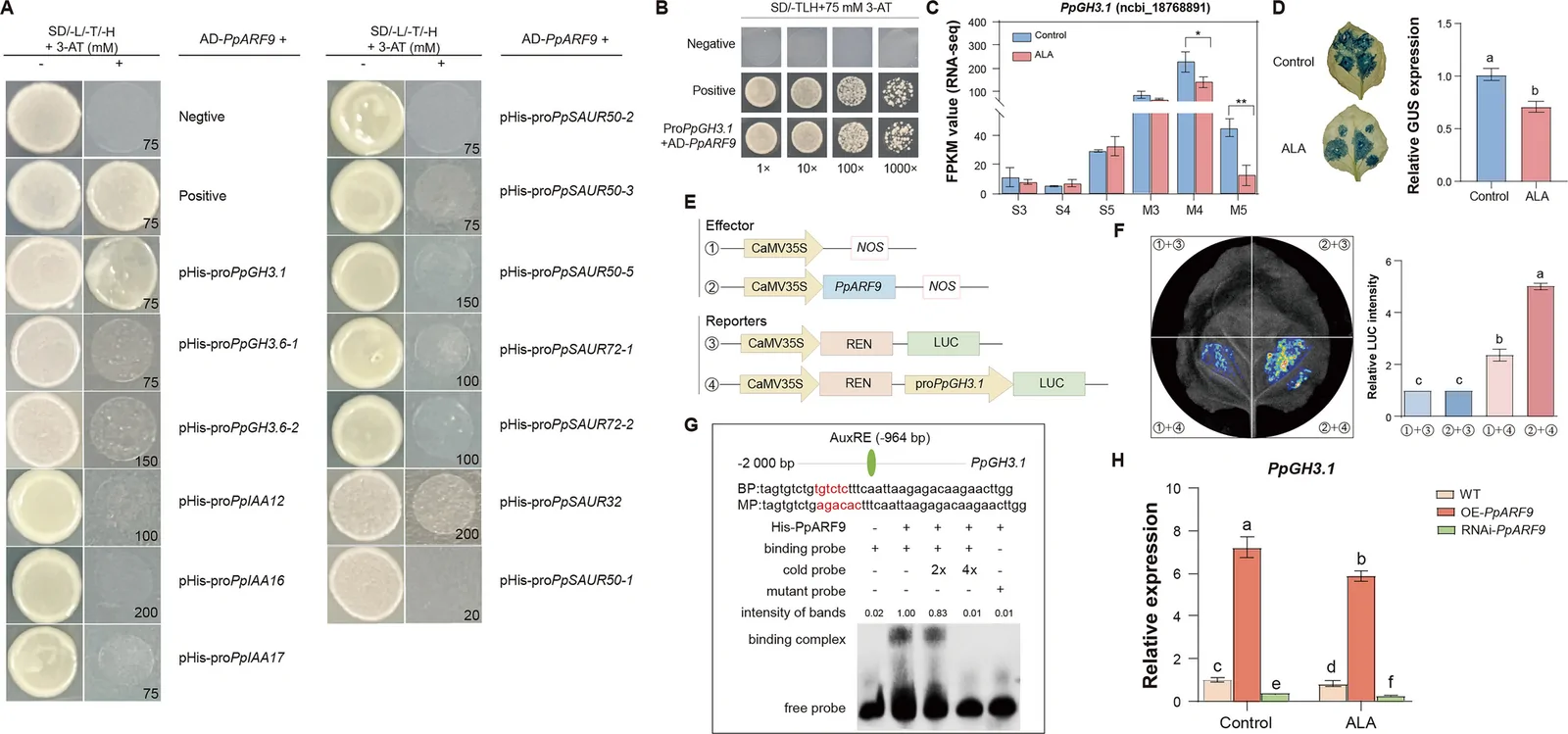
Transcriptional regulation of PpARF9 on the downstream gene *PpGH3.1*. **A** Yeast one-hybrid assay (Y1H) for PpARF9 binding to the promoters of 13 early auxin response DEGs (*IAA*/*AUX*, *GHs*, *SAURs*) selected from the transcriptome, which contain AuxRE in the promoters and are possibly involved in auxin response. The differential expression and function description of 13 DEGs are listed in Table S4. SD/-L–T-H indicates synthetic dropout media lacking leucine, tryptophan, and histidine. Meanwhile, pGADT7 and pHIS2 mixed plasmid was used as the negative control, pGADT7-Rec2-53 and pHIS2-p53 were the positive control. The numbers in the lower right corner are the 3-AT (3-Amino-1,2,4-triazole) concentrations. **B** Identification of PpARF9 binding to promoter of *PpGH3.1* by Y1H. The yeast was cultured on SD/-L–T-H solid media containing 75 mM 3-AT, with different dilutions. **C** Transcription level of *PpGH3.1* by means of FPKM value from RNA-seq data. S represents seeds; M represents mesocarps. 3, 4, 5 represent the third, fourth and fifth week after ALA spraying. Asterisks '*' and '**' indicate significance at *P* = 0.05 and 0.01, respectively. **D** GUS staining of pro*PpGH3.1*-GUS transiently injected into tobacco leaves treated with ALA. Three biological replicates were performed with a representative image presented here. The relative expression of pro*PpGH3.1*-GUS in the tobacco leaves is presented on the right, which are the means of three biological replicates, and the different small letters represent significant difference at *P* = 0.05. **E** Schematic diagrams of effector and reporter vector for the DLR assay. **F** Detection of the LUC signal in tobacco leaves (left) and measurement of relative LUC intensity in the assay (right). Representative image based on three replicates. **G** EMSA was performed to verify the binding of protein PpARF9 to the *PpGH3.1* promoter in vitro. “ + ” or “ − ” indicates the presence or absence of the proteins or probes in the reaction system. The red letters represent the AuxRE (TGTCTC) and mutant element (AGACAC). **H** Relative expression of *PpGH3.1* in the WT, OE-*PpARF9*, RNAi-*PpARF9* peach callus cell lines treated with 1.0 mg L^−1^ ALA or not, which are described in Fig. 6G. The data are means ± SE of three biological replicates. The different small letters represent significant differences at *P* = 0.05

To investigate how PpARF9 regulates downstream gene expression, we identified 13 early auxin responsive DEGs in the transcriptome (Table S4), which harbor the auxin response element (AuxRE, TGTCTC) in the promoters and are possibly involved in auxin response. Yeast one-hybrid (Y1H) assay showed that PpARF9 failed to bind the promoters of 12 genes including *PpGH3.6–1*/−2, *PpIAA12*/*16*/*17*, *PpSAUR50-2*/*−3*/*−5*, and *PpSAUR72-1*/*−2*/*32*/*50–1* (Fig. 7A). The only exception was the promoter of *PpGH3.1*. After suppressing self-activation with 75 mM 3-AT, yeast colonies containing *PpARF9* and *PpGH3.1* promoter grew well even at a 1000-fold dilution (Fig. 7B). These indicate that *PpGH3.1* may be the target gene of PpARF9. RNA-seq data showed that ALA had no influence on the *PpGH3.1* transcript levels in the seeds of peach, but significantly declined the expression in the mesocarps from the 4th and 5th week after ALA spraying (Fig. 7C). A pro*PpGH3.1*-GUS reporter assay in tobacco leaves revealed weaker GUS staining under ALA treatment, and RT-qPCR corroborated reduced GUS transcript abundance (Fig. 7D). These demonstrate that ALA represses the *PpGH3.1* promoter activity. In dual-luciferase reporter (DLR) assay, co-expression of *PpARF9* with the *PpGH3.1* promoter increased LUC signal by 2.1-fold (*P* < 0.01; Fig. 7E and F). EMSA further demonstrated that PpARF9 specifically bound to a 40-bp probe encompassing the AuxRE of *PpGH3.1* promoter in vitro. Binding was abolished by an unlabeled competitor or by mutating TGTCTC to AGACAC (Fig. 7G). In transgenic peach callus, *PpGH3.1* transcript levels were higher in OE-*PpARF9* lines and lower in RNAi-*PpARF9* lines compared to the WT. Exogenous ALA reduced *PpGH3.1* expression both in OE-*PpGH3.1* and RNAi-*PpGH3.1* callus. These findings suggest that *PpGH3.1* expression is upregulated by PpARF9 but downregulated by ALA (Fig. 7H). Therefore, as a downstream gene of PpARF9, *PpGH3.1* expression is negatively regulated by ALA.

**Fig. 8:**
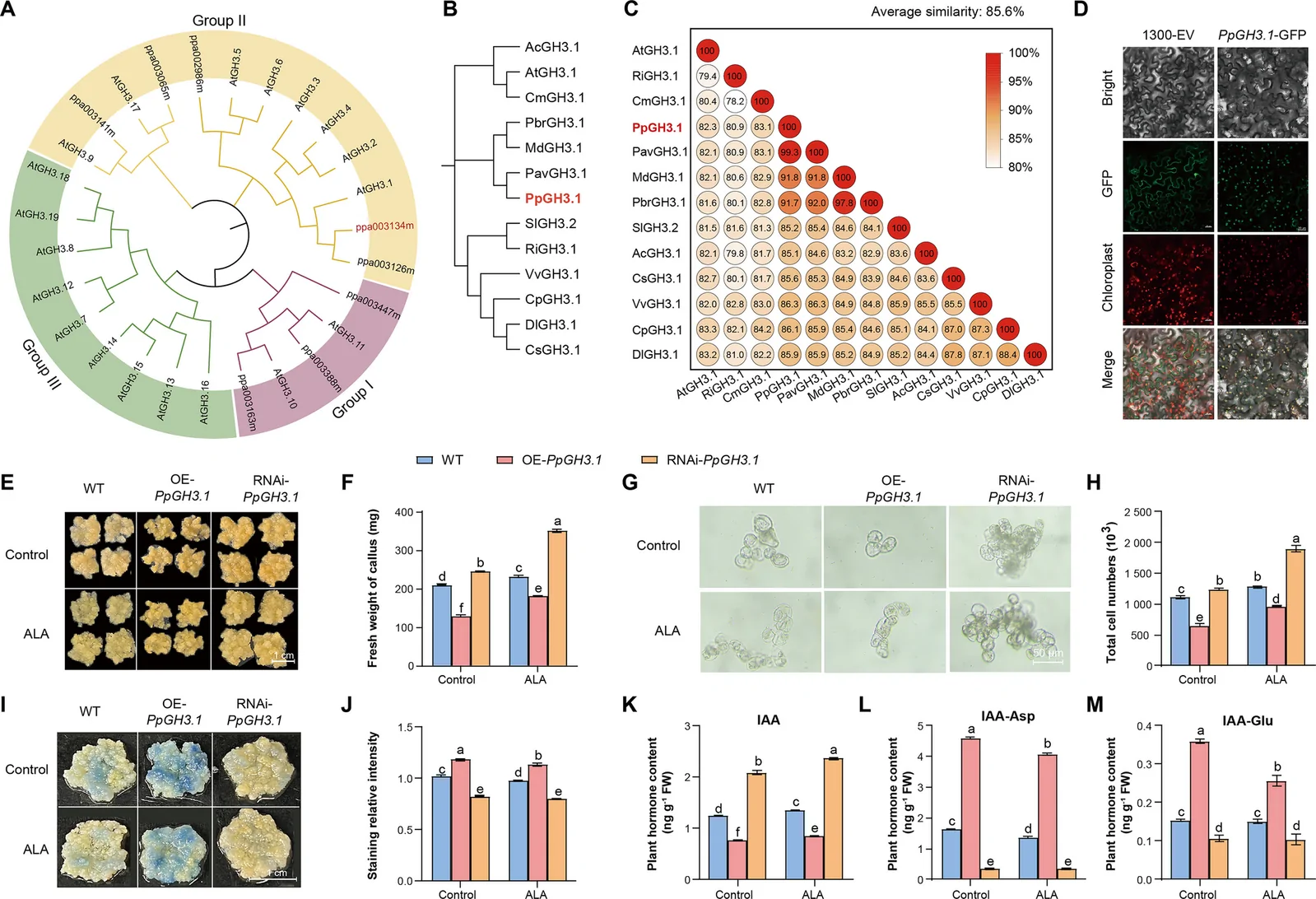
Functional identification of PpGH3.1 in ALA-regulated cell division in peach callus. **A** Phylogenetic relationship of PpGH3 with AtGH3 proteins of *Arabidopsis thaliana*. **B** Phylogenetic analysis using amino acid sequences of PpGH3.1 and other 12 species GH3.1 members, including kiwifruit (*Actinidia chinensis*), *Arabidopsis* (*A. thaliana*), melon (*Cucumis melo*), pear (*P. bretschneideri*), apple (*M. domestica*), sweet cherry (*Prunus avium*), peach (*P. persica*), tomato (*S. lycopersicum*), raspberry (*Rubus idaeus*), grape (*V. vinifera*), papaya (*Carica papaya*), longan (*Dimocarpus longan*), and orange (*Citrus sinensis*). **C** Heatmap of sequence alignment similarity using amino acid sequences of PpGH3.1 and other species GH3.1 members. The numbers and colors within circles indicate the percentage of similarity. **D** The subcellular localization of PpGH3.1 protein. All fluorescence images were acquired on a ZEISS LSM 900 laser-scanning confocal microscope (Carl Zeiss AG, Oberkochen, Germany). The emission wave of GFP and chloroplast were collected at 495–530 and 575–630 nm. The scale bar = 20 μm. **E–F** Callus phenotypes and fresh weight of 'ZJB' peach, which was stably transformed with overexpression (OE-*PpGH3.1*) and RNA interference (RNAi-*PpGH3.1*) vectors. The fresh weights of callus are listed in the right histogram. The scale = 1 cm. The positive identification of the transgenic cells by PCR and RT-qPCR was shown in Fig. S5. **G-H** Observation of cell suspension under a microscope and cell number counter in the right histogram (the scale = 50 μm). **I-J** Detection of cell viability by Evans blue staining and relative intensity is listed in the right histogram. **K-M** Content of IAA, IAA-Asp, IAA-Glu in transgenic callus of WT, OE-*PpGH3.1*, and RNAi-*PpGH3.1,* cultured in MS medium with 1.0 mg L^−1^ ALA or not. The data are means ± SE of three independent replicates. The different small letters in each panel represent significant differences at *P* = 0.05

Phylogenetic analysis of 27 GH3 proteins (19 from *Arabidopsis* and 8 from peach) showed PpGH3.1 (Ppa003134m) is situated with AtGH3.1 in the Group II (Fig. 8A). The orthologues of GH3.1 from 13 species share > 85% amino-acid identity (Fig. 8B and C), implying functional conservation of the genes. Subcellular localization of a PpGH3.1-GFP fusion in tobacco leaves exhibited chloroplast signals (Fig. 8D). To ascertain the functions, we generated OE-*PpGH3.1* and RNAi-*PpGH3.1* peach callus, which were verified by PCR and RT-qPCR (Fig. S5). After 15 d culture on MS medium, the fresh weight of OE-*PpGH3.1* callus was 38% lower than that of the WT, whereas that of RNAi-*PpGH3.1* callus was 17% higher than the latter (*P* < 0.01; Fig. 8E and F). ALA treatment significantly increased callus size in all lines but did not fully rescue the OE callus. Cell suspensions derived from the callus revealed similar trends: OE-*PpGH3.1* contained 41% fewer cells, whereas RNAi-*PpGH3.1* did 12% more cells, than the WT (Fig. 8G and H). Evans blue staining showed lower viability in OE-*PpGH3.1* and higher viability in RNAi-*PpGH3.1*. ALA alleviated these effects but not in RNAi-*PpGH3.1* (Fig. 8I and J). The observed null effect of ALA on cell viability in RNAi-*PpGH3.1* genotypes provides genetic evidence that PpGH3.1 serves as the essential downstream component required for ALA-promoted fruit development. Quantification of auxin metabolites showed that OE-*PpGH3.1* callus contained much less free IAA but more IAA-Asp and IAA-Glu than the WT, whereas RNAi-*PpGH3.1* exhibited the opposite profile. Furthermore, exogenous ALA increased the free auxin content but decreased the conjugate content in all genotypes (Fig. 8K-M). These results suggest that PpGH3.1 catalyzes conjugation of IAA.

Furthermore, we conducted RT-qPCR to detect expression of cell cycle related genes with *PpGH3.1* transgenic callus cell lines (Fig. S4B). Compared with the WT, the majority of cell cycle genes, including *PpDPa*, *PpCYCA3-4*, *PpCYCB1-2*/*2–3*, *PpCYCD3-3*, and *PpCDKA*/*B2,* were significantly downregulated in OE-*PpGH3.1* callus, whereas the expression of *PpRBR* and *PpMYB3R3* was substantially upregulated. An opposite expression pattern for these genes was observed in RNAi-*PpGH3.1* callus. These results indicate that *PpGH3.1* is intimately associated with cell cycle regulation and modulates the cell division rate. ALA significantly elevated the expression of most cell cycle genes, such as *PpE2F, PpDPa*, *PpCCSA52A2*, *PpMYB3R1*, *PpCYCA2-4*/*3–2*/*3–4*, *PpCYCB1-2*/*1–4*, *PpCYCD3-3*/*4–1*/*5–1*, and *PpCDKA*/*B1*/*B2*, but concurrently repressed *PpRBR* and *PpMYB3R3* across all genotypes (*P* < 0.05). These suggest that ALA treatment modulates the cell cycle, at least in part, through the *PpGH3.1*-mediated gene expression. And *PpE2F*, *PpDPa*, *PpRBR*, *PpMYB3R3*, *PpCYCA3-4*, *PpCYCB1-2*, *PpCYCD3-3,* and *PpCDKA* are responsive to both ALA and *PpGH3.1*. Collectively, our data demonstrate that *PpGH3.1* is a direct downstream target of *PpARF9*. The indole-3-acetic acid amide synthetase encoded by *PpGH3.1* reduces free auxin levels via amidation. Together, *PpGH3.1* and *PpARF9* negatively regulate ALA-induced mesocarp cell division in peach fruit. RT-qPCR analyses further confirm that the two genes collaboratively regulate the cell cycle, with *PpDPa*, *PpRBR*, *PpMYB3R3* and *PpCYCD3-3* serving as key candidate targets. Both factors act as negative regulators of cell division, and ALA alleviates their inhibitory effects by downregulating *PpGH3.1* and *PpARF9* expression.

## Discussion

### ALA promotes young peach fruit development

For decades, ALA has been recognized to boost crop yields across diverse species, such as kidney bean, barley, radish, garlic, potato (Hotta et al. 1997), wheat (Al-Thabet 2006), and carrot (Xu et al. 2023). These suggest that ALA, a natural plant growth substance can be widely used in agriculture (Wang et al. 2023a). ALA is also known to improve the fruit quality of external appearance and flavor in apple (Wang et al. 2004), grape (Watanabe et al. 2006; Xie et al. 2013a), jujube (Guo et al. 2010), strawberry (Cheng et al. 2012), litchi (Feng et al. 2015) peach (Guo et al. 2015), and pear (Liu et al. 2022). Among them, the mechanisms for ALA to promote anthocyanin accumulation has been well demonstrated recently (Fang et al. 2022; Zhang et al. 2025; Yin et al. 2025). Comparatively, how ALA improves the other quality of fruits is not well studied. In date palm, ALA was observed to promote the fresh weight and fruit sizes (Al-Khateeb et al. 2006). In peach, ALA enhances single fruit weight by 18.59% with the increases of longitudinal and transverse diameter by 11.98% and 5.37%, respectively (*P* < 0.05) (Guo et al. 2015). However, it was also reported that ALA spraying five weeks after flower falling improved the flavor quality of mature peach but not fruit mass (Ye et al. 2017). Consequently, the potential of ALA can improve peach fruit size remains elusive. In current study, we applied ALA solution to the rhizosphere of peach trees one week before blossom for the first time to increase chilling resistance against the late spring cold (Yuan et al. 2025), and foliar spraying after full blossoming for the second time to improve fruit development (Liang et al. 2023), the results indicated that both fruit size and flavor quality were promoted at maturity (Fig. 1; Fig. S1). It is consistent with the previous study in pear (Shen et al. 2011), in which foliar application of ALA at late bloom increased fruit weight by 23.65%. In grape, ALA spraying one week after full blooming, the single berry weight was increased by 26%−37% at mature (Xie et al. 2013a). However, ALA did not promote peach fruit enlargement when applied 5 weeks after flower falling (Ye et al. 2017). The reason for this may be spraying too late, missing the cell division period of fruits. According to our research, the benefits of early ALA application are manifold. For example, ALA application at sprouting stage (Zhang et al. 2022) or before blossom (Yuan et al. 2025) can increase leaf photosynthesis and resistance to chilling stress. In another study, ALA application at the early stage enables leaves synthesizing more carbohydrates, facilitate to improve fruit quality (Liang et al. 2023). Similarly in current study, we observed that the ALA promoted peach fruit sizes including the fresh weight as well as longitudinal and transverse diameters (Fig. 1B). Although the increases in the first two weeks after foliar spraying were modest, the differences became significant thereafter. Therefore, in order to increase fruit size, ALA should be applied as early as possible.

### ALA negatively regulates PpARF9 expression to promote cell division in peach fruits

In the peach genome, 17 *PpARF* members were identified (Diao et al. 2020), among which, *ppa002617m* was designated as *PpARF12*. In our transcriptome data, a gene with the ID of ncbi_18776765 can be named as *PpARF9* when the *Arabidopsis ARF* family served as the reference. However, alignment of two sequences showed that our *PpARF9* and the *PpARF12* in the former study (Diao et al. 2020) are identical. Since the NCBI RefSeq transcriptome was employed in our sequencing pipeline, we retain the name *PpARF9* to facilitate cross-study comparison. The sequences of AFR9 are highly conserved across species (Fig. 6A and B). We used the complete ARF protein sets, including 23 of *A. thaliana*, 21 of tomato (*S. lycopersicum*), 17 of peach (*P. persica*), 16 of sweet cherry (*P. avium*), 12 of woodland strawberry (*F. vesca*), 17 of cucumber (*C. sativa*) and 59 of soybean (*G. max*) to construct a bigger phylogenetic tree (Fig. S6, the sequence-acquisition metadata for *ARF* gene families of seven species is listed in Table S5). The results shows that the ARF members can be divided into 4 classes, where all ARF9 homologs fall in Class I, consistent with the previous reports (Okushima et al. 2005; Wu et al. 2011; Hou et al. 2021). PpARF9 is very close to PavARF9, FvARF9, and CsARF9b. It is also close to GmARF9-1/−2, SlARF9, and AtARF9, suggesting that all ARF9 may possess similar biological functions. In tomato, *SlARF9* is highly expressed in the early stages of fruit development and negatively regulates cell division (De-Jong et al. 2015). In cucumber, *CsARF9* inhibits embryo growth and seed filling during young seed development (Liu et al. 2025b). In soybean, *GmARF9b* negatively regulates cortical cell layers and the longitudinal cell length of roots, thereby inhibiting root growth (Li et al. 2025a). In the woodland strawberry, *FvARF9* is involved in the regulation of fruit development and maturation (Wang et al. 2019). In sweet cherry, *PavARFs* are involved in the regulation of fruitlet abscission. Among them, *PavARF9* contains the most ACGTG motifs related to ABA response, suggesting that it might play a role in the ABA signaling pathway (Hou et al. 2021).

In our study, the expression levels of *PpARF9* in both seeds and mesocarps of peach fruits were decreased from the 3rd to 5th week after ALA spraying, which was further decreased by ALA treatment (Fig. 4B and C). Hormone association analysis revealed that *PpARF9* expression was strongly negatively correlated with the IAA content (Fig. 4D and E). These imply that *PpARF9* may be involved in IAA catabolism. We transformed OE- and RNAi-*PpARF9* vectors into ‘ZJB’ peach callus, the results showed that PpARF9 negatively regulated cell growth, cell number and cell activity (Fig. 6G-I). The GUS staining assay showed that ALA depressed the transcriptional activity of the *PpARF9* promoter (Fig. 6E), which aligns with the results in the transgenic cell lines that ALA alleviated the depression of OE-*PpARF9* but strengthened the promotion of RNAi-*PpARF9* on cell proliferation. Since localized in the nucleus (Fig. 6F), it is reasonable to deduce that PpARF9 may function as an ALA-responsive transcriptional repressor involved in the negative regulation of cell division in peach fruits.

In tomato, NOR-like1 (a NAC transcription factor) can bind to the *SlARF9* promoter, activating its transcription and resulting in fewer cell layers and smaller fruits (Peng et al. 2023). We did not study the upstream regulatory factor of *PpARF9* but selected a potential downstream target gene in current study (Fig. 7). The results of Y1H showed that PpARF9 can bind to the promoter of *PpGH3.1* but not to the other auxin-responsive genes, including *PpGH3.6–1*/3.6–2, *PpIAA12*/*16*/17, *PpSAUR50-2*/50–3*/50–5*/*72–1*/72–2/*32*/*50–1*, even though all these genes were identified as the DEGs in the transcriptome (Table S4). In the transcriptome, ALA significantly depressed *PpGH3.1* expression in the mesocarps of peach fruits after ALA spraying 4th and 5th weeks (Figs. 4B and 7C). Further DLR and GUS assay showed that PpARF9 positively regulated the transcriptional activity of the *PpGH3.1* promoter (Fig. 7E and F) while ALA negatively regulated the transcriptional activity (Fig. 7D). There is a cis-element AuxRE in *PpGH3.1* promoter. EMSA showed that PpARF9 can specifically bind to this element (Fig. 7G). Furthermore, in the transgenic callus of peach, OE-*PpARF9* promoted *PpGH3.1* expression, while RNAi-*PpARF9* significantly depressed the gene expression. In addition, exogenous ALA generally aggravated the depression (Fig. 7H). Collectively, *PpGH3.1* is a downstream target gene of PpARF9 involved in ALA-regulated processes.

Functional redundancy and synergistic or antagonistic interactions between ARF family members are widespread across land plants (Chandler 2016). In *Arabidopsis*, repressors AtARF1/2 share redundant functions with activator AtARF6 in the regulation of embryonic cell division (Rademacher et al. 2011). In tomato, the paralogous pair SlARF2A/2B exhibits functional redundancy to synergistically control fruit ripening via auxin-ethylene crosstalk, with dual-gene silencing causing far more severe defects than single-gene silencing (Hao et al. 2015). Similarly, SlARF5, SlARF7, SlARF8A and SlARF8B act redundantly and synergistically to regulate tomato fruit growth (Hu et al. 2023). In peach, previous work has suggested that PpARF2B, PpARF10A and PpARF12 may function synergistically to drive root growth (Diao et al. 2020), yet the potential synergistic or antagonistic interactions between PpARF9 and other PpARF members remain uninvestigated, especially in the context of ALA-regulated fruit development. In this study, our transcriptome data showed that only *PpARF9* exhibited significant differential expression under ALA treatment, with no significant transcriptional changes detected for the other 16 *PpARF* members (Table S6). We further performed in silico protein–protein interaction prediction between PpARF9 and other PpARFs using AlphaFold 3 (Abramson et al. 2024). The results (Table S7) showed that none of the other PpARFs met the widely accepted high-confidence interaction criteria (ipTM > 0.6, pTM > 0.5). This suggests that these PpARF proteins are unlikely to physically interact with PpARF9. Taken together, *PpARF9* is the sole member of the *PpARF* family which is involved in mediating ALA-regulated peach fruit development.

ARF-Aux/IAA protein interaction is the core of canonical auxin signal transduction. Cancé et al. (2022) resolved the structural basis of this interaction: both ARF and Aux/IAA proteins contain a conserved PB1 domain enabling head-to-tail PB1-PB1 binding via electrostatic forces and hydrogen bonds. Numerous studies have confirmed the key regulatory role of ARF-Aux/IAA protein interaction in plant growth and organ development. In apple, MdIAA29 directly interacts with MdARF4 at the protein level to inhibit its transcriptional regulation of downstream *GH3* genes (Lei et al. 2024). MdARF-MdIAA interaction pairs regulate fruit development via auxin signal transduction (Zhou et al. 2021). In *Populus*, PtoIAA9 interacts with PtoARF5 via C-terminal III/IV domains to regulate the xylem development (Xu et al. 2019). In *Arabidopsis*, Goh et al. (2012) revealed that multiple Aux/IAA-ARF modules cooperatively regulate lateral root formation. We constructed a PPI network using DEGs between the control and ALA groups (Fig. S8). The results showed that PpARF9 has a tight interaction association with PpIAA12/16/17 and PpAUX22D. Further studies should prioritize verifying the direct interactions between PpARF9 and the candidate PpIAA/AUX proteins in vitro and in vivo.

### PpGH3.1 in chloroplasts attenuates ALA-promoted mesocarp cell division via auxin conjugation

Three gene family members including *auxin/indole-3-acetic acid* (*AUX*/*IAA*), *gretchen hagen3* (*GH3*), and *small auxin-up RNA* (*SAUR*) are called as the early auxin response genes (Bao et al. 2024). Among them, *GH3* encodes auxin amide synthetase (Luo et al. 2023). In peach, 8 GH3 proteins can be classified into two groups, where GH3.1 belongs to Group II, sharing with AtGH3.1. Unlike *Arabidopsis*, the peach genome lacks Group III GH3 proteins (Fig. 8A). It is known that *PpGH3.1* expression is responsive to naphthylacetic acid (NAA) and the enzyme is responsible for conjugation of free auxin with amino acids into inactive compounds (Zeng et al. 2015). In the lateral root (LR) primordia, *Atgh3.1*/*2*/*3*/*4*/*5*/*6* hextuple (*Atgh3hex*) mutants display a higher LR density due to increased LR initiation and faster LR development, implying that GH3 affects meristematic activity (Wang et al. 2023b). In apple, *MdGH3* genes respond to abiotic stresses (Yuan et al. 2013), while melon *CmGH3* is involved in fruit development (Chen et al. 2023a). In longan (*Dimocarpus longan*), *DlGH3.1* is associated with pericarp growth (Kuang et al. 2011), while *Citrus sinensis CsGH3.1* enhances resistance to citrus canker (*Xanthomonas citri* subsp. *citri*) by attenuating auxin signaling (Zou et al. 2019). In other fruits, such as papaya (Liu et al. 2016), grape (Böttcher et al. 2010), raspberry (Bernales et al. 2019), kiwifruit (Gan et al. 2019) and tomato (Sravankumar et al. 2018), *GH3.1* orthologues regulate fruit ripening.

In the current study, we found that *PpGH3.1* expression in the mesocarps of peach was greatly decreased from the 4th week to 5th week after ALA spraying, and exogenous ALA further downregulated the gene expression (Fig. 7C). When the OE-*PpGH3.1* vector was transformed into ‘ZJB’ peach callus, the cell growth was significantly depressed with less cell number and lower cell viability than that of the WT. Conversely, when the RNAi-*PpGH3.1* vector was transformed, all of these indices were reversed (Fig. 8E-J). Meanwhile, OE-*PpGH3.1* decreased the endogenous auxin levels but increased IAA-Asp and IAA-Glu content, whereas RNAi-*PpGH3.1* had the opposite effect. Moreover, exogenous ALA inhibited the effect of OE-*PpGH3.1* but improved that of RNAi-*PpGH3.1* (Fig. 8K-M). Collectively, *PpGH3.1* is an ALA-responsive gene, transcriptionally regulated by PpARF9, responsible for IAA conjugation, negatively affecting cell division via auxin depletion in peach mesocarps. It is consistent with a previous report that rice OsARF19 transcriptionally regulated *OsGH3-5* expression to delete free auxin and affected plant architecture (Zhang et al. 2015).

The subcellular localization of GH3 proteins is diverse. OsGH3-5 is localized at the endoplasmic reticulum (ER) in rice (Zhang et al. 2015), while sugarcane (*Saccharum hybrid*) ShGH3-1 is in both the plasma membrane and the nucleus (Zou et al. 2022), and pea (*Pisum sativum*) PsGH3 is predominantly cytosolic (Ostrowski et al. 2013). AtFIN219, a GH3-like protein in *Arabidopsis*, is a cytoplasmic protein (Hsieh et al. 2000), whereas pepper (*Capsicum annuum* L.) CaGH3-5 and CaGH3-7 are dually targeted to the plasma membrane and chloroplasts (Zang et al. 2025). These suggest that GH3 family proteins exhibit diverse subcellular localization with diverse functions. In current study, we found peach PpGH3.1 localized in chloroplast (Fig. 8D), where free IAA is conjugated with amino acids to maintain IAA homeostasis and regulates different physiological processes (Hayashi et al. 2021).

### ALA-PpARF9-PpGH3.1 regulatory module affects cell division through cell cycle associated gene expression

The cell cycle, a fundamental process for eukaryotic propagation, is composed of four phases, including G1, S, G2, and M (Gombos et al. 2023). The progression of cell cycle is strictly governed by the DREAM complex and CyclinA/B/D-CDKA/B modules, where the DREAM complex is composed of DP, RBR, E2F, APC/C and MYB3Rs transcription factors (Shimotohno et al. 2021). Different components are encoded by multiple genes, positively or negatively affecting the cell cycle progression. In *Arabidopsis*, the DPb-E2Fc heterodimer is a repressor, which can be degraded via ubiquitination after CYCD-CDKA dependent phosphorylation. Auxin enhances DPb-E2Fc degradation and promotes G1/S transition. In contrast, the DPa-E2Fb is an activator-type heterodimer, which can be stabilized by auxin, and promotes cell cycle (Gombos et al. 2023). In peach, EARLY BUD BREAK 1 (EBB1), an ERF transcription factor, can promote bud break. When OE-*PpEBB1* was heterologously transformed into poplar, the auxin, ABA, and TZ cytokinin levels are significantly increased compared with the WT. Many DEGs associated with cell cycle occurred. Among them, *PpCYCD1.1*/*2.1*/*3.2/3.3* are upregulated expression, whereas *PpCYCA1.2*, *PpCYCB1.1*/*1.3*, *PpCYCD6.1*, and *PpCYCP2.1* downregulated expression (Zhao et al. 2020). It seems that most of *PpCYCDs* are the positive while the others are the negative for cell cycle.

In the current study, OE-*PpARF9* or OE-*PpGH3.1* in peach callus significantly downregulated genes such as *PpDPa* and *PpCYCD3-3* expression but upregulated such as *PpRBR* and *PpMYB3R3* expression. Conversely, RNAi-*PpARF9* or RNAi-*PpGH3.1* produced the opposite expression pattern. Furthermore, exogenous ALA strengthens the effects of both OE- and RNAi- but the direction is opposite (Fig. S4). Additionally, PpARF9 was found directly targeting AuxREs in cell-cycle genes (Fig. S7). Therefore, we propose that *PpARF9* and *PpGH3.1* cooperatively suppress the G1/S transition by repressing *PpDPa* and *PpCYCD3-3* expression while enhancing the accumulation of the repressors *PpRBR* and *PpMYB3R3*, thereby concurrently blocking G1-S progression and ultimately reducing the cell-division rate. When ALA is added, the repressor gene expression is inhibited, while the activator gene expression is promoted. Consequently, cell cycle progression is accelerated, leading to increased mesocarp cell proliferation in peach fruit. Yet, the detailed mechanisms for *PpARF9* and *PpGH3.1* to regulate gene expression associated with cell cycle warrant further investigation.

**Fig. 9:**
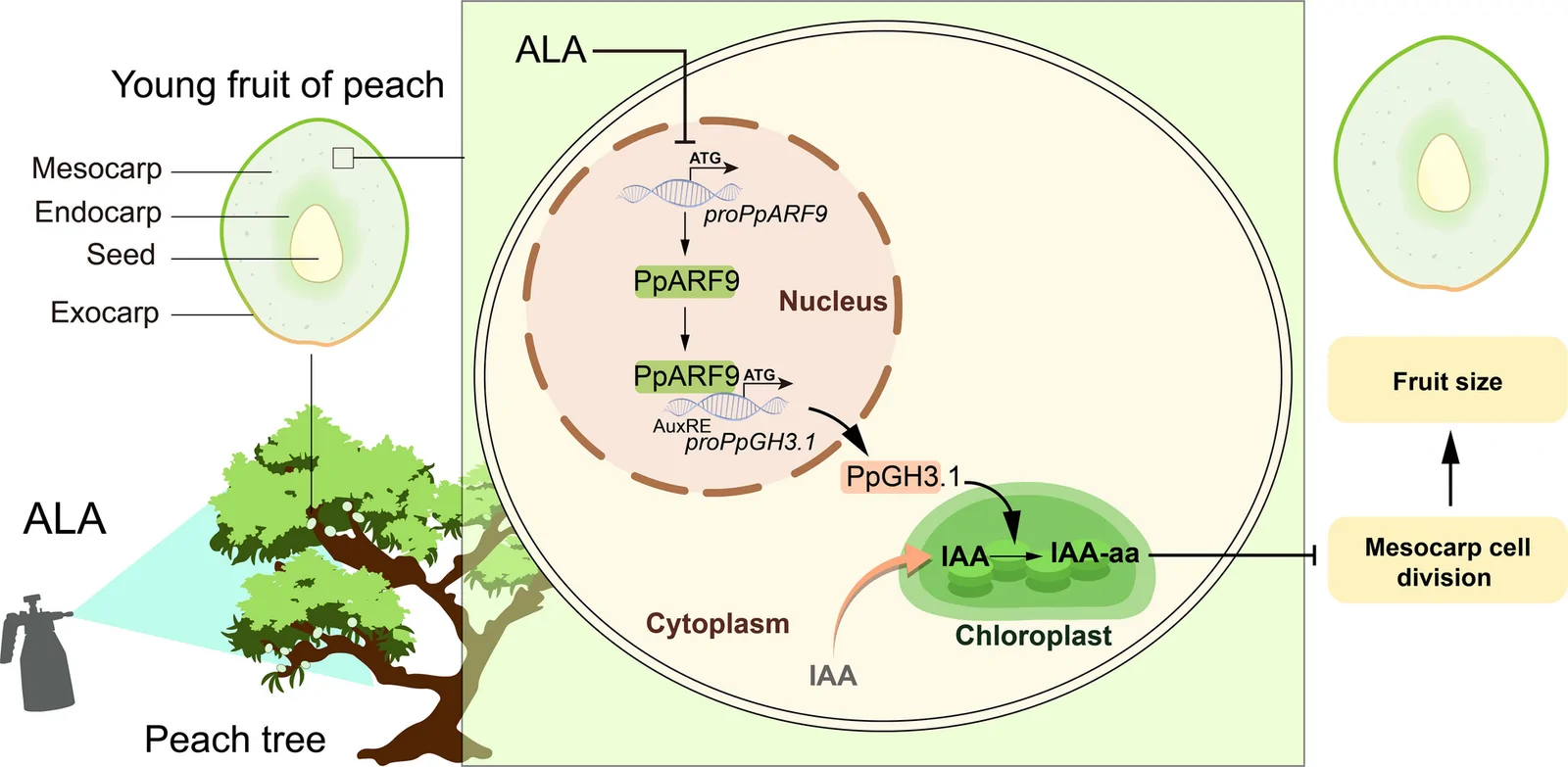
A working model for ALA to promote young fruit development of peach. Following ALA treatment, the transcription of *PpARF9* is repressed, thereby blocking its transcriptional activation of *PpGH3.1*. Consequently, free auxin accumulates in the mesocarp, stimulating cell division and ultimately contributing to peach fruit enlargement. The arrows represent positive regulation whereas the flat heads represent negative regulation

In summary, we propose a working model for ALA to promote peach fruit size in Fig. 9. It preliminarily elucidates how exogenous ALA application inhibits the expression of *PpARF9* and *PpGH3.1* genes, alleviates the inactivation of IAA conjugating to amino acids within chloroplasts, maintains elevated IAA levels, thereby promoting cell division in pericarp cells, increasing cell number, and facilitating the development of larger fruit size and final quality. Nevertheless, since it is the first report to explore the molecular mechanism of ALA in promoting fruit growth, there are many issues needed to be further addressed. In addition, cell division in young fruits begins from pollination and fertilization (He and Yamamuro 2022), then, ideally, samples should have been collected from the first week after ALA foliar spraying. However, no sufficient seeds could be sampled for multiple omics analysis at the early stage. Therefore, we had to sample from the 3rd week. Although the results well demonstrated the promotive effect of ALA and unveiled the underlying mechanisms, we can modify the sampling time in the future. Given the high evolutionary conservation of ARF9 and GH3.1 across species (Figs. 6A–C and 8B–C) and ALA can effectively enlarge fruit sizes across species (Wang et al. 2025), the ALA-PpARF9-PpGH3.1 regulatory module identified in this study may represent a broadly applicable mechanism for improving fruit size in other fruit crops, such as apple, pear, and tomato through the modulation of auxin-regulated cell division. This mechanism warrants thorough and detailed investigation.

## Materials and methods

### Plant materials and treatments

The field trial was conducted in 2022 with four-year-old peach trees (*P. persica* var. *nectarina* cv. Zhongyou 4), grown in a density of 1 × 4 m with a “Y” shape and long shoot pruning in the Nanjing Doukou Agricultural Technology Company, Jiangsu Province, China. Sixty trees with uniform status were selected and randomly assigned to two groups (the ALA treatment and the control) after winter pruning. One week before blossom, the ALA-treated trees were applied with 5 L of 10 mg L^−1^ ALA solution to the rhizosphere while the controls with the same amount of clean water. When 50% flowers were opened, approximately 30 flowers that opened the day were marked on a single tree with a marker pen to ensure that all the fruits picked during the experiment were formed from the flowers that opened on the same day. Two days later, the 30 trees of ALA treatment were foliar sprayed with 10 mg L^−1^ ALA solution with a mist projector, while the remaining 30 trees with clean water. One week after, the labeled young fruits were randomly sampled at one-week interval. At each sampling, three biological replicates were set for the ALA treatment and the control, and each sample contained ten fruits. The morphological indices, including single fruit weight, vertical and horizontal diameter were measured after sampling. The fruits for paraffin section were fixed in the FAA (formaldehyde: glacial acetic acid: 70% alcohol = 1: 1: 18) solution. The fruits for hormone or transcriptome analysis were dissected into mesocarps and seeds, which were immediately frozen in liquid nitrogen and stored at −80 °C refrigerator for further analysis.

### Preparation of paraffin sections and microscopical observation of peach fruits 3–5 weeks after ALA spraying

During the experiment, we found that fruit sizes between two treatments were obviously different from the 3rd week after ALA spraying, then, the paraffin sections were prepared using the young fruits 3–5 weeks after ALA spraying. Before fixation, they were horizontally and vertically sliced along the central axis using a sharp blade, then the slices were submerged in the FAA solution more than 24 h to fix the cellular architecture. The section preparation including alcohol-dehydration, xylene-clearing, wax-infiltration and embedding, slicing, de-waxing, and staining, followed the method of former study (Mao 2013). The sections about 8 μm thickness were examined under an Olympus fluorescent inverted microscope.

To select the maximum cross-sectional section, the different sections of the same fruit were carefully checked under the microscope with 40 × magnification. Based on comparison of the length, width, area, seed status, and distribution of main vascular bundles across different sections, the maximum section of the fruit was identified, which was further photographed under 400 × magnification into about 100 vision fields, then pieced together into a complete picture of the fruits by Photoshop software. The cell area and cell number in the mesocarps, endocarp and seed of a fruit were randomly measured by the built-in scale bar, calculated by ImageJ software. The area of a single cell = total local area/the cell number (> 100) in the region, and the cell number in different fruit parts = the area/the single cell number of mesocarps, endocarps and seeds, respectively. All data were the means of 10–20 regions of three fruits.

### Detection of plant hormones in the young fruits

Peach fruits were collected 3–5 weeks after ALA spraying, which were divided into mesocarps and seeds, and separately ground into powder under liquid nitrogen. Approximately 1 g of the powder was put into a test tube, and 10 mL acetonitrile and 2 µL internal standard solution (10 ng mL^−1^) was added sequentially. The mixture was incubated at 4 °C for 2 h, then centrifuged at 12 000 g for 5 min. The supernatant was collected. The sediment was extracted twice more and the supernatant were pooled. The mixture was added with 35 mg of C_18_ packing material, then shaken thoroughly for 30 s, centrifuged at 10 000 g for 5 min. The supernatant was collected and evaporated to dryness with a nitrogen flow, and the residue was redissolved in 400 µL methanol. The solution was filtered through a 0.22 µm organic filter membrane, and 2 µL of the filtrate was injected into a high-performance liquid chromatography-tandem mass spectrometer (HPLC–MS/MS, ABQtrap6500) for detection. The quantitative and qualitative ions were listed in Table S8. The mobile phase was composed of A (methanol/0.1% formic acid) and B (water/0.1% formic acid). Standard curves were constructed using standards at concentrations of 0.1, 0.2, 0.5, 2, 5, 20, 50, and 200 ng mL^−1^. Each data of a sample was mean of three biological replicates.

### Transcriptome sequencing and differentially expressed genes (DEGs) identification

Total RNA of the mesocarps and seeds of peach fruits sampled during 3–5 weeks after ALA spraying was extracted using TRIzol™ reagent (Invitrogen, Carlsbad, CA, USA) according to the manufacturer’s protocol. RNA quality was assessed on an Agilent 2100 Bioanalyzer (Agilent Technologies, Palo Alto, CA, USA) with RNase free agarose gel electrophoresis. Then, the mRNA was enriched by Oligo(dT) beads, fragmented into short fragments using fragmentation buffer and reversely transcribed into cDNA by using NEBNext Ultra RNA Library Prep Kit for Illumina (NEB #7530, New England Biolabs, Ipswich, MA, USA). The purified double-stranded cDNA fragments were end repaired, A base added, and ligated to Illumina sequencing adapters. The ligation reaction was purified with the AMPure XP Beads (1.0 ×). Ligated fragments were subjected to size selection by agarose gel electrophoresis and PCR amplification. The resulting cDNA library was sequenced using Illumina Novaseq6000 by Gene Denovo Biotechnology Co. (Guangzhou, China).

The quality of raw reads was controlled by FASTP (Chen et al. 2018). Reads containing adapters, with an N ratio greater than 10%, or composed entirely of A bases were filtered to obtain clean reads. Hisat2 (Kim et al. 2015) was used to align the clean reads to the peach reference genome (GCF_000346465.2). Transcript reconstruction was performed using StringTie (Pertea et al. 2015). RSEM (Li et al. 2011) was employed to calculate gene expression levels, expressed as raw read count and Fragments Per Kilobase of exon model per Million mapped fragments (FPKM) values. Principal component analysis (PCA) was conducted using R (http://www.r-project.org/) to assess the reproducibility of biological replicates. Differentially expressed genes (DEGs) were analyzed with DESeq2 (Love et al. 2014). Gene with *P*-value < 0.05 and |Fold change|> 1.5 were identified as significantly DEGs (Zhu et al. 2022). These DEGs were mapped to the Kyoto encyclopedia of genes and genomes (KEGG) (https://www.kegg.jp/) to analyze metabolic pathways enriched by DEGs.

### Determination of quality of mature peach fruits

Ten weeks after ALA spraying, twelve mature fruits were harvested from each treatment. Four of them from each treatment made up a replicate, which were ground into a homogenate and three biological replicates were prepared. The anthrone colorimetric method was used to determine the soluble sugar content (Zhang et al. 2020). The Coomassie brilliant blue method was used to determine the soluble protein content (Sedmak et al. 1977). Sodium hydroxide titration was used to determine titratable acid content. An assay kit (Beijing Biolead Biotech. Co., Ltd.) was used to determine vitamin C (VC) content with the colorimetric method. All measurements were conducted with three biological replicates, and represented by the means and standard errors.

### Real-time quantitative polymerase chain reaction (RT-qPCR)

The RNA extracted from peach fruits was reverse-transcribed into cDNA following the instruction of the TransScript (One-Step gDNA Removal and cDNA Synthesis Supermix, TransGen Biotech, Beijing, China). The RT-qPCR reaction system was prepared using the SYBR Green Premix *Pro Taq* HS qPCR (Accurate Biotechnology (Hunan) Co., Ltd, Changsha, China) Kit (Rox Plus) and performed on a QuantStudio5Flex real-time PCR (ABI, Los Angeles, CA, USA). The primers for RT-qPCR were designed using TBtools and synthesized by Tsingke (Table S9). *PpTEF* (encoding translation elongation factor) was used as the internal reference gene of peach (Tong et al. 2009). Relative transcript levels were calculated using the 2^−ΔΔCT^ method.

### Phylogenetic analysis and multiple sequence alignments

Amino acid sequences of different species were obtained from NCBI (https://www.ncbi.nlm.nih.gov/) and TAIR (https://www.arabidopsis.org/). Eleven ARF9s and 13 GH3s were used to construct phylogenetic trees, respectively, by the neighbor-joining method (bootstrap = 1 000 replicates) to analyze the kinship by DNAMAN software. Multiple sequence alignment of ARF9 and GH3 homologues from different plant species was performed using Clustal Omega (https://www.ebi.ac.uk/jdispatcher/msa/clustalo?outfmt=fa). Conserved motifs were analyzed by MEME (https://meme-suite.org/meme/tools/meme) and visualized through TBtools.

### Assay of the effect of ALA on the transcriptional activity of the gene promoters of peach

About 2 000 bp upstream of promoters of *PpARF9* and *PpGH3.1* were cloned and inserted into the pBI121 vector at *Xba* I and *Hin*d III sites, replacing the 35S promoter to construct the recombinant vectors. *Agrobacterium* harboring the recombinant plasmids was cultured until OD_600_ reached 0.8. The culture liquid was then centrifuged at 5 000 rpm for 10 min, resuspended in a buffer containing 10 mM 2-morpholino ethanesulfonic acid (MES), 10 mM MgCl_2_, 100 μM Acetosyringone (AS) and incubated at 28 °C with shaking at 200 rpm for 1 h. Four-week-old tobacco (*Nicotiana benthamiana*) with fully expanded leaves were used for *Agrobacterium*-mediated infiltration. The bacterial suspension was injected into leaves using a 1 mL syringe until the leaf surface appeared moist. After infiltration, the leaves were sprayed with 0.5 mg L^−1^ ALA solution. The plants were kept in the dark at 24 °C for 2 d, followed by 1 d light exposure. For GUS staining, the infiltrated leaves were incubated in GUS staining solution, containing 100 mM phosphate buffer (pH = 8.5), 1 mM 5-bromo-4-chloro-3-indolyl B-D-glucuronide (X-Gluc), 0.1% (v/v) Triton X-100, 10 mM ethylene diamine tetraacetic acid (EDTA), 0.5 mM K_4_[Fe (CN)_6_], 0.5 mM K_3_[Fe (CN)_6_], at 37 °C for 24 h. Then, they were washed with 70% (v/v) ethanol and imaged under a Leica M165C microscope (Germany). In addition, the injected tobacco leaves were collected for RT-qPCR of *GUS* expression, with *NtActin* as the internal reference gene.

### Genetic transformation in ‘ZJB’ peach callus

The coding sequence (CDS) of *PpARF9* and *PpGH3.1* were ligated and inserted into the pBI121 vector at the sites of *Xba* I and *Bam*H I to transform the recombinant plasmid into *Agrobacterium* GV3101. To silence *PpARF9* and *PpGH3.1*, specific fragments of the *PpARF9* and *PpGH3.1* CDS were cloned and recombined into the pHELLSGATE4 vector (abbreviated as P4) to construct RNA interference plasmids (RNAi-*PpARF9*, RNAi-*PpGH3.1*). The primers for vector construction were listed in Table S10. *Agrobacterium* containing the recombinant plasmids was cultured until OD_600_ ≈ 0.8. Two-week-old ‘Zaojiubao’ (‘ZJB’) peach callus, kindly granted by Professor Cao of Hebei Agricultural University, were immersed in the *Agrobacterium* suspension and shaken at 200 rpm for 30 min at 28 °C. The callus was then co-cultivated on MS medium, containing 1.0 mg L^−1^ 6-benzylaminopurine (6-BA), 0.2 mg L^−1^ indole butyric acid (IBA), 0.1 mg L^−1^ gibberellic acid 3 (GA_3_) for 2 d (Wang et al. 2024a). Then, they were transferred to the screening MS medium, containing 1.0 mg L^−1^ 6-BA, 0.2 mg L^−1^ IBA, 0.1 mg L^−1^ GA_3_, 300 mg L^−1^ cephalosporins (Cep), 80 mg L^−1^ kanamycin (Kana) or 25 mg L^−1^ Hygromycin (Hyg) at 25 °C in the dark for 30 d. When the new cells occurred on the old callus, they were verified by PCR and RT-qPCR (Zhang et al. 2025). Then, the positive cells were cultured on MS solid media in a chamber with 100 μmol m^−2^ s^−1^ photon flux density, 16 h light/8 h dark and 25 °C. To test the effect of ALA on cell proliferation, 1.0 mg L^−1^ ALA was added into the medium after filtration sterilization. Fifteen days later, the phenotypes of different genotypic callus of peach were observed and the fresh weight was weighted.

To assess the role of different genotypes on cell numbers, the above callus was resuspended in 10 mL of MS liquid medium, repeatedly aspirated with a pipette to form free cell suspension until callus clumps disappeared. Taking 20 μL of the suspension to a clean glass slide and covering with a coverslip, the free cells were observed under an optical microscope (10 x) and calculated with the cell counter plugin of ImageJ (V1.53t). Each sample was randomly observed 10 fields, and three biological samples were repeated.

To assess the role of different genotypes on cell viability, Evans blue staining was used (Vijayaraghavareddy et al. 2017). One milliliter of 0.5% (w/v) Evans blue solution (Coolaber SL7200, Beijing, China) was applied to immerse the callus surface, then incubated in the dark for 24 h at 25 °C. After the supernatant discarded, the callus was gently rinsed with sterile deionized water three times (5 min each). The callus was placed evenly on a sterile petri dish and photographed at fixed distances under standardized lighting conditions using a digital camera (RAW format, 300 dpi). The staining area percentage was determined using the Otsu threshold method of ImageJ, with pixel grayscale values to represent relative staining intensity. The darker the staining, the lower the cell viability is. The test was repeated three times.

### Assay of subcellular localization of PpARF9 and PpGH3 proteins

The subcellular localization of PpARF9 and PpGH3 proteins were predicted using the Cell-PLoc 2.0 website based on their amino acid sequences. Furthermore, the CDS of these genes without stop codons was amplified and recombined with the linearized pCambia 1300 plasmid vector at *Bam*H I and *Xba* I sites via homologous recombination. *Agrobacterium* containing the recombinant plasmid was cultured at 28 °C. An infection solution was prepared by resuspending and activating the *Agrobacterium*, with pCambia 1300 empty vector (EV) as a control. The infection solution was injected into the leaves of 4-week-old *N. benthamiana* using a 1 mL medical syringe. After 36 h of dark incubation and 24 h of light exposure, the mesophyll tissue near the injection site was collected. A confocal microscopy (LSM900, Zeiss, Germany) was used to detect green fluorescent protein (GFP), chloroplast fluorescence. Image acquisition and processing were performed using ZEN 2012 software.

### Assay of yeast one-hybrid (Y1H)

The promoters of *PpGH3s*, *PpIAAs*, and *PpSAURs* were separately cloned into the pHIS2 vector. The full length of *PpARF9* was inserted into the pGADT7 vector to construct pGADT7-*PpARF9*, pGADT7 and pHIS2 mixed plasmid was used as negative control, pGADT7-Rec2-53 and pHIS2-p53 were the positive control. Y187 yeast cells were co-transformed with these reconstructed vectors. The co-transformants were selected on SD/-Leu/-Trp/-His medium supplement with 3-amino-1,2,4- triazole (3-AT) to assess PpARF9 binding to the promoters of the downstream genes.

### Assay of dual-luciferase reporter (DLR)

The CDS of *PpARF9* was cloned into the pBI121 vector to generate the effector vector, with the empty vector as a negative control. The promoter of *PpGH3.1* was cloned into pGreenII0800-LUC to generate the reporter vectors. These reporters were individually transformed into *A. tumefaciens* GV3101 (pSoup). Mixtures of bacteria expressing different combinations of reporters and effectors were injected into tobacco leaves. After 60 h, the LUC signal was detected using a PIXIS 1024B system (United States).

### Electrophoretic mobility shift assay (EMSA)

The CDS of *PpARF9* was cloned into the pET32a T-1 vector to generate His-PpARF9 fusion proteins. The His-PpARF9 proteins were expressed in *Escherichia coli* BL21 (DE3) cells induced by 0.5 mM isopropyl β-D-thiogalactoside (IPTG) and purified using a His-tag protein purification kit (Beyotime, P2226, Beyotime Biotech. Shanghai, China). The EMSA probes were labelled with biotin at the 5′-end and the EMSA was performed using a chemiluminescent EMSA kit (Beyotime, GS009, Beyotime Biotech. Shanghai, China). All primer sequences for vector construction were listed in Table S10.

### Statistical analysis

All data were the means of at least three biological replicates. Statistical analyses were performed using SPSS software (22.0, USA) to analyze one-way and two-way analysis of variance (ANOVA) and assess the difference significance, followed by Tukey's honestly significant difference test or Duncan's multiple range test. When two groups of data were compared, Student's *t*-test was performed. When *P* < 0.05, the difference is considered as significant. Picture collation was conducted by GraphPad Prism, ImageJ, and Photoshop.

## Supplementary Information

Supplementary Material 1.

Supplementary Material 2.

## Data Availability

The data will be available from the corresponding author upon reasonable request.

## Abbreviations

ABA: Abscisic acid
ALA: 5-Aminolevulinic acid
ARF: Auxin response factor
AuxRE: Auxin responsive element
CDS: Coding sequence
CTK: Cytokinin
DEGs: Differentially expressed genes
DLR: Dual-luciferase reporter assay
EMSA: Electrophoretic mobility shift assay
FPKM: Fragments per kilobase of transcript per million mapped reads
GA: Gibberellic acid
GFP: Green fluorescent protein
GH3: Gretchen Hagen3
IAA: Indole-3-acetic acid
KEGG: Kyoto Encyclopedia of Genes and Genomes
OE: Over expression
RNAi: RNA interference
RT-qPCR: Real-time quantitative polymerase chain reaction
WT: Wild type
Y1H: Yeast one-hybrid assay
