# Supplementary material for: 5-Aminolevulinic acid promotes cell division and young fruit development by suppressing the PpARF9–PpGH3.1 auxin-conjugation module in peach
Source: Mol Hortic. 2026 Sep 7;6:69. doi: 10.1186/s43897-026-00262-7 (PMC13548669; doi:10.1186/s43897-026-00262-7)
Supplement: Supplementary file 1 [file 43897_2026_262_MOESM1_ESM.docx]

**Fig. S1 Fruit quality at harvest after ALA treatment. (A)** Comparison of mature fruits between the ALA treatment and the control (n = 12), the scale = 5 cm. The line shows that ALA-treated fruits are bigger than that of the control. **(B)** The content of soluble sugars, soluble solids, titratable acids, soluble proteins, vitamin C, and sugar-acid ratio in the mature fruits, respectively. The different letters indicate the significant difference (*P* < 0.05).

**Fig. S2 Correlation analysis of RT-qPCR with FPKM (RNA-seq).** Eight differentially expressed genes (DEGs) were randomly selected from the transcriptome dataset and validated by RT-qPCR. In the figure, S and M denote seed and mesocarp, respectively; the numerals 3, 4 and 5 indicate the third, fourth and fifth week after exogenous ALA spraying. The X-axis displays the combination of tissue and weeks after ALA spraying. The left Y-axis shows the relative expression level determined by RT-qPCR (2^-ΔΔCT^), while the right Y-axis shows the absolute transcript abundance derived from RNA-seq (FPKM value). Pearson correlation analysis (two-tailed) was used to evaluate the consistency of each gene between the two methods.

**Fig. S3 Positive identification of OE-*PpARF9* and RNAi-*PpARF9* calli. (A)** Genomic DNA was extracted from calli that over-expressed or silenced *PpARF9* and analyzed by agarose gel electrophoresis using a 2-kb DNA ladder. A distinct band at the expected size (~2.0 kb) was taken as evidence of successful transformation. Three independent cell lines were characterized for *PpARF9* overexpression and RNAi construct. **(B)** The relative transcript level of *PpARF9* was quantified by RT-qPCR with *PpTEF* as the internal reference. Expression values were calculated using the 2^–ΔΔCT^ method. Data are presented as mean ± standard error of at least three biological replicates. Groups labeled with different lowercase letters indicate significant difference (one-way ANOVA followed by Duncan’s multiple-range test, *P* < 0.05).

**Fig. S4 Heatmap of relative expression levels of cell-cycle-related genes in transgenic peach callus tissue. (A)** Relative expression levels of cell-cycle-related genes in the *PpARF9* transgenic peach callus (including WT, OE-, and RNAi-) treated with ALA or not. **(B)** Relative expression levels of cell-cycle-related genes in the *PpGH3.1* transgenic peach callus (including WT, OE-, and RNAi-). The values are log₂-transformed normalized expression levels. The color scale ranges from blue (low) to red (high). Row labels indicate genotypes and treatments, and column labels indicate genes involved in cell cycle.

**Fig. S5 Positive identification of OE-*PpGH3.1* and RNAi-*PpGH3.1* calli. (A)** Genomic DNA was extracted from cell lines that over-expressed or silenced *PpGH3.1* and analyzed by agarose gel electrophoresis. Recombinant plasmids pCAMBIA1302-*PpGH3.1* and pHELLSGATE4-*PpGH3.1* served as positive controls (+), and an equal volume of sterile ultrapure water (-) was used as the negative control. A distinct band at the expected molecular weight was taken as evidence of successful transformation. Three independent cell lines were selected and validated for both *PpGH3.1* over-expression and RNAi- vectors. **(B)** The relative transcript abundance of *PpGH3.1* in the three genotypes (wild-type, over-expression, and RNAi lines) was quantified by RT-qPCR with *PpTEF* as the internal reference. Expression levels were calculated using the 2^–ΔΔCT^ method. For each genotype, three biological replicates were performed. Data are presented as mean ± SEM. Different lowercase letters indicate significant differences (one-way ANOVA followed by Duncan’s multiple-range test, *P* < 0.05).

**Fig. S6 Analysis of phylogenetic tree of full-length ARF proteins from seven species.** A total of 165 ARF members were used to construct the phylogenetic tree, including 23 of *Arabidopsis thaliana*, 21 of tomato (*Solanum lycopersicum*), 17 of peach (*Prunus persica*), 16 of sweet cherry (*Prunus avium*), 17 of cucumber (*Cucumis sativus*), 12 of woodland strawberry (*Fragaria vesca*) and 59 of soybean (*Glycine max*). Multiple sequence alignment was performed with MEGA X using MUSCLE. An unrooted neighbor-joining tree was constructed (1,000 bootstrap replicates; p-distance model; pairwise deletion) and visualized with iTOL (<https://itol.embl.de>). Colored circles at the branch tips indicate the source species as illustrated in the inset legend. The tree was partitioned into four colored sectors to highlight the four major ARF class (I–IV) inferred from clade topology. PpARF9 is marked in red color.

**Fig. S7 EMSA analysis reveals that PpARF9 binds to the AuxRE elements within the promoters of *PpCCS52A1* (A), *PpDPa* (B) and *PpCYCD3-3* (C)**. EMSA reactions were carried out using only the corresponding binding probes. The 2000 bp upstream promoter of *PpCCS52A1* contains four AuxREs, while *PpDPa* and *PpCYCD3-3* possess three and two AuxREs (tgtctc), respectively. Since the two cis-acting elements AuxRE-1 and AuxRE-2 are tightly tandemly repeated within the promoter of *PpCCS52A1*, the binding probe (BP1) was designed as tgtctctgtctc for EMSA. The symbol ‘+’ indicates presence, and ‘−’ indicates absence.

**Fig. S8 Protein–protein interaction (PPI) analysis of PpARF9 with Aux/IAA family proteins.** PPI networks of target proteins are constructed by utilizing the interaction relationships from the STRING protein-protein interaction database (http://string-db.org). The analysis was performed on all DEGs identified from the comparison between the ALA treatment and the control. The network illustrates the interaction connections of PpARF9 with other proteins. Node size represents gene expression abundance. Interaction strength is indicated by the binding score, which can be visualized by node color or edge thickness. The redder or thicker, the higher binding score. Since the binding scores of all these interacting pairs reached 999, the node colors and edge thicknesses are uniform across the network. NCBI_18767398 (Aux22D): auxin - induced protein 22D; NCBI_18793200 (AUX22D): auxin - induced protein IAA4; IAA12/16/17 corresponds to auxin - responsive protein IAA12/16/17. ABP19A denotes auxin - binding protein 19a and UBI11 denotes polyubiquitin.
